# Effective factors of improved helmet use in motorcyclists: a systematic review

**DOI:** 10.1186/s12889-022-14893-0

**Published:** 2023-01-05

**Authors:** Pouya Mahdavi Sharif, Sara Najafi Pazooki, Zahra Ghodsi, Ahmad Nouri, Hamed Abbasizade Ghoroghchi, Reza Tabrizi, Mehdi Shafieian, Seyed Taghi Heydari, Rasha Atlasi, Mahdi Sharif-Alhoseini, Alireza Ansari-Moghaddam, Gerard O’Reilly, Vafa Rahimi-Movaghar

**Affiliations:** 1 Sina Trauma and Surgery Research Center, Tehran University of Medical Sciences, Tehran, Iran; 2 School of Medicine, Tehran University of Medical Sciences, Tehran, Iran; 3 Brain and Spinal Cord Injury Research Center, Neuroscience Institute, Tehran University of Medical Sciences, Tehran, Iran; 4 Faculty of Medicine, Semnan University of Medical Sciences, Semnan, Iran; 5 Noncommunicable Diseases Research Center, Fasa University of Medical Sciences, Fasa, Iran; 6 The Department of Biomedical Engineering, Amirkabir University of Technology (Tehran Polytechnic), Tehran, Iran; 7 Health Policy Research Center, Institute of Health, Shiraz University of Medical Sciences, Shiraz, Iran; 8 Endocrinology and Metabolism Research Center, Endocrinology and Metabolism Clinical Sciences Institute, Tehran University of Medical Sciences, Tehran, Iran; 9 Health Promotion Research Center, Zahedan University of Medical Sciences, Zahedan, Iran; 10 Department of Epidemiology and Preventive Medicine, School of Public Health and Preventive Medicine, Monash University, Melbourne, Australia; 11 Universal Scientific Education and Research Network (USERN), Tehran, Iran; 12 Institute of Biochemistry and Biophysics, University of Tehran, Tehran, Iran; 13 Visiting Professor, Spine Program, University of Toronto, Toronto, Canada

**Keywords:** Helmet, Head protective devices, Motorcycles, Wounds and injuries, Accidents, Traffic, Systematic review

## Abstract

**Background:**

Road traffic injuries (RTI) are one of the most prominent causes of morbidity and mortality, especially among children and young adults. Motorcycle crashes constitute a significant part of RTIs. Policymakers believe that safety helmets are the single most important protection against motorcycle-related injuries. However, motorcyclists are not wearing helmets at desirable rates. This study systematically investigated factors that are positively associated with helmet usage among two-wheeled motorcycle riders.

**Methods:**

We performed a systematic search on PubMed, Scopus, Web of Science, Embase, and Cochrane library with relevant keywords. No language, date of publication, or methodological restrictions were applied. All the articles that had evaluated the factors associated with helmet-wearing behavior and were published before December 31, 2021, were included in our study and underwent data extraction. We assessed the quality of the included articles using the Strengthening the Reporting of Observational Studies in Epidemiology (STROBE) checklist for observational studies.

**Results:**

A total of 50 articles were included. Most evidence suggests that helmet usage is more common among drivers (compared to passengers), women, middle-aged adults, those with higher educations, married individuals, license holders, and helmet owners. Moreover, the helmet usage rate is higher on highways and central city roads and during mornings and weekdays. Travelers of longer distances, more frequent users, and riders of motorcycles with larger engines use safety helmets more commonly. Non-helmet-using drivers seem to have acceptable awareness of mandatory helmet laws and knowledge about their protective role against head injuries. Importantly, complaint about helmet discomfort is somehow common among helmet-using drivers.

**Conclusions:**

To enhance helmet usage, policymakers should emphasize the vulnerability of passengers and children to RTIs, and that fatal crashes occur on low-capacity roads and during cruising at low speeds. Monitoring by police should expand to late hours of the day, weekends, and lower capacity and less-trafficked roads. Aiming to enhance the acceptance of other law-abiding behaviors (e.g., wearing seat belts, riding within the speed limits, etc.), especially among youth and young adults, will enhance the prevalence of helmet-wearing behavior among motorcycle riders. Interventions should put their focus on improving the attitudes of riders regarding safety helmets, as there is acceptable knowledge of their benefits.

**Supplementary Information:**

The online version contains supplementary material available at 10.1186/s12889-022-14893-0.

## Background

Road traffic injuries (RTIs) are responsible for more than 1.3 million deaths worldwide, which makes them the 8^th^ most prevalent cause of mortality among the total population and the first cause among children and adults aged 5–29 years [1]. Motorcycle riders constitute 28% of these deaths worldwide; however, this statistic varies considerably between different World Health Organization (WHO) regions. For instance, in the South-East Asia region, 43% of road traffic deaths are among motorcycle riders, and this proportion is 36% for the Western Pacific region and 23% for the Americas region [2]. According to the U.S. Centers for Disease Control and Prevention (CDC) statement, the establishment of a universal helmet law is the single most effective strategy to tackle the issue of motorcyclists’ deaths and associated costs [3]. Wearing a motorcycle helmet can alleviate the risk of death and head injuries by approximately 42% and 69%, respectively [4].

Despite the establishment of legislation and public awareness campaigns, the prevalence of helmet usage is generally low among motorcycle riders, especially in East Asian countries [5, 6], where the helmet usage rate is generally lower than 50%, and in some instances, it is only 3% [7]. It can be inferred from this fact that the sole enaction of mandatory helmet laws is not conducive to enhancing helmet-wearing behavior among motorcycle riders. Since there has been no comprehensive study in the literature to gather the effective factors for helmet usage among motorcyclists, we performed this systematic review to reach this aim. To our knowledge, until now, there is not any published study that has comprehensively addressed factors that positively influence helmet usage among motorcycle riders. The findings of this study will shape the direction of upcoming interventions to identify the susceptible sub-populations more precisely and avoid spending resources on less-effective approaches.

## Methods

### Outcome definition

This systematic review aimed to collect, critically appraise, and synthesize all available data on factors that are positively associated with the safety helmet-wearing behavior among users of two-wheeled, motorized motorcycles.

### Search strategy and study selection

A systematic search was performed by an expert librarian (RA) in this field using the following databases: PubMed, Scopus, Embase, Web of Science, and the Cochrane Central Register of Controlled Trials (CENTRAL). We carried out the database search, screening of papers, data extraction, and research synthesis according to the Preferred Reporting Items for Systematic Reviews and Meta-Analyses (PRISMA) guidelines [8]. The search was conducted using keywords from relevant studies, medical subject headings (MeSH) terms, and experts’ opinions in this field. We have provided the search strategy that we have applied for each database in Supplementary Table 1. No limitations regarding language, publication date, or methodologic class were applied. All relevant articles that had been published before December 31, 2021, were retrieved for further screening. The reviewers manually searched the reference lists of the included articles to identify relevant missing studies. All articles that identified and discussed the positive factors associated with safety helmet usage among motorized motorcycle riders were included. To assess the quality of the included articles, the Strengthening the Reporting of Observational Studies in Epidemiology (STROBE) checklist for observational studies was used [9]. This quality assessment tool comprises 22 items under introduction, methods, results, discussion, and funding domains [9]. Although the quantification of the STROBE is discouraged by some authors [10], in compliance with most published articles, we assigned one score to each of the 22 items if they were fulfilled by the article, with a sum score ranging between 0 and 22. Accordingly, any article that fell below the 50^th^ percentile was recognized as low quality [11]. Four trained investigators (PMS, SNP, AN, and HAG) screened papers and performed the data extraction and quality assessment in two independent groups. The process of screening and data extraction was supervised by another two authors (ZG and VR). Disagreements were resolved by consensus.

### Data extraction

The amassed data were entered into a previously prepared data extraction form. The following variables were searched within each article:

1. Study characteristics, i.e., the characteristics of the first author, year and location of the conduction of the study, and methodological design.
2. The size of the target population and related demographic characteristics (including, but not limited to, age, sex, educational attainments, occupation and income level, marital status, type of residence, riding position specifications, religion, type of residence, etc.).
3. Motorcycle characteristics (e.g., engine size, license possession, ownership of helmets, etc.).
4. Motorcycle trip-related factors (e.g., the distance, frequency, and purpose of travel, plus the timing of travel within the day and week, type of roads traveled, history of previous crashes, climate conditions during riding, alcohol consumption before riding, etc.).
5. Knowledge, attitude, and practices regarding safety helmet usage and reasons for their application.

The investigators were instructed to extract any relevant data not included in the data extraction form and insert it in a separate column(s). Any variable that has been investigated by the included papers is discussed in this systematic review (Tables 1 and 2 and Supplementary Table 2).

**Table 1:** Characteristics of included studies and assessed variables for their potential correlations with helmet-wearing behavior

First author	Year of conduction	Location	Design	Sample size and characteristics	Assessed factors	Associated factors	STROBE
Ackaah [12]	2010	Tamale metropolis, Ghana	Cross-sectional (observational)	3115 drivers and 1058 passengers	Age (estimated), sex, driving status, riding location	Age (estimated), driving status, riding location	17
Merali [13]	2010–2014	Cambodia	Cross-sectional (observational)	62,039 child passengers 12 years of age and younger (apparent age)	Helmet wearing by the driver, province, day of the week, time of the day, no of passengers, no of children	Helmet wearing by the driver, province, day of the week, time of the day, no of passengers	20
Mirkazemi [14]	2008–2009	Pune city, India	Cross-sectional (questionnaire)	2259	Helmet ownership, sex, age, marital status, education, socioeconomic class, type of family, and type of residence	sex, marital status, type of family, and type of residence	19
Adnan [15]	2017	Karachi, Pakistan	Repeated cross-sectional (questionnaire)	246 and 277	Age, license possession, number of daily trips, education riding experience, history of severe crashes, the experience of a stolen helmet, marital status, monthly income, length of trips, time of the day, mean fuel consumption, enforcement campaign	Age, license possession, number of daily trips, monthly income, riding experience, history of crashes, the experience of a stolen helmet, marital status, license possession, number of daily trips, and age in the CART model	18
Aghamolaei [16]	Not mentioned	Bandar-Abbas, Iran	Cross-sectional (questionnaire)	221	TBP and HBM constitutes	Perceived behavioral control, perceived barriers, self-efficacy, and cues to action	20
Akaateba [17]	Not mentioned	Wa, Ghana	Cross-sectional (observational)	11,360 drivers and 3107 passengers	Sex, time of the day, day of the week, riding location, driving status	Sex, time of the day, time of the week, riding location, driving status	19
Akaateba [18]	2013	Wa, Ghana	Cross-sectional (observational and questionnaire)	271	Sex, age, marital status, occupation, education, license possession, helmet ownership, knowledge, attitude, and beliefs	Sex, age, marital status, occupation, attitude, and belief	19
Aidoo [19]	2017	Kumasi, Ghana	Cross-sectional (survey)	503	Sex, age, marital status, education, occupation, frequency of driving, law awareness, helmet ownership, history of crashes, license possession, riding experience, length of trips	Sex, marital status, education, helmet ownership, license possession	16
Babio [20]	1999	Spain	Cross-sectional (survey)	1029	Age, sex, education, income, marital status, community size, type of roads, occupation, social class	Age, income, community size, type of roads, education	17
Ranney [21]	2006–2008	USA	Cross-sectional (web-based survey)	445 newly licensed motorcyclists	Age sex, education, marital status, history of previous injuries, license possession, type of motorcycle, attitudes, and beliefs	Sex, education, history of previous injuries, attitudes, and beliefs	20
Bachani [22]	2010–2011	Cambodia	Cross-sectional (observational and questionnaire)	68,894 observations and 304 interviews	Time of the day, driving status, attitude, helmet ownership	Time of the day, driving status, attitude	18
Bao [23]	2011–2014	Vietnam	Cross-sectional (observational and questionnaire)	301,981 observations and 2730 interviews	KAP and reasons for using helmets	KAP and reasons for using helmets	13
Skalkidou [24]	1998	Greater Athens, Greece	Cross-sectional (questionnaire)	982 riders and 349 passengers	Sex, age, driving status, day of the week, time of the day, type of roads, type of motorcycle, riding experience, license possession, attitude	Sex, driving status, day of the week, time of the day, type of roads, type of motorcycle, attitude	15
Ghasemzadeh [25]	2014	Charoymag (rural areas), Iran	Cross-sectional (observational and questionnaire)	150	TPB, subjective norms, attitude, age, economic status, license possession, education, occupation, history of crashes, driving status	TPB, subjective norms, attitude, economic status, education, occupation, driving status	19
Wadhwaniya [26]	2011–2013	Hyderabad, India	Cross-sectional (observational and questionnaire)	4774 drivers and 97 passengers	Sex, age, education, driving status, type of motorcycle, motorcycle ownership, the purpose of the trip, helmet ownership	Sex, age, education, driving status, type of motorcycle, the purpose of the trip, helmet ownership	20
Hernández [27]	2013	Colombia	Cross-sectional (observational and questionnaire)	3980	Sex, age, climate condition, type of roads, socioeconomic status	Sex, type of roads, socioeconomic status	18
Hung [28]	2005–2006	Hai Duong Province, Vietnam	Cross-sectional (questionnaire)	716 drivers and 92 passengers	Sex, age, education, income, driving status, type of roads, type of motorcycle, type of residence, driving frequency, driving experience, driving distance, license possession, occupation, alcohol consumption, attitudes	Age, education, income, driving status, type of roads, driving distance, attitudes	20
Jiwattanakulpaisarn [29]	2009	Thailand	Cross-sectional (questionnaire)	2429 drivers and 1328 passengers	Sex, age, driving frequency, attitudes	Sex, age, driving frequency, attitudes	15
Khan [30]	Not mentioned	Karachi, Pakistan	Cross-sectional (questionnaire)	300 male drivers	Age, education, marital status, knowledge about enforcement laws	Education	18
Kumphong [31]	Not mentioned	Khon Kaen, Thailand	Cross-sectional (observational)	27,977 drivers and 6,947 passengers	Age (apparent age), sex, driving status, type of motorcycle, time of the day, day of the week, no of passengers, presence of police, red-light running behavior	Age (apparent age), sex, driving status, type of motorcycle, time of the day, day of the week, no of passengers, presence of police, red-light running behavior	17
Sreedharan [2]	2007	Kerala state, India	Cross-sectional (questionnaire)	309	Sex, age, education, marital status, religion, alcohol consumption, attitudes	Sex, age, marital status, alcohol consumption, attitudes	17
Shults [32]	2011	USA	Cross-sectional (survey and interview)	211 youths (aged 12–17 years)	Sex, driving frequency, location of residence	Driving frequency	19
Xuequn [33]	2009	Zhongshan, Guangdong Province, China	Cross-sectional (observational)	13,410 drivers and 4498 passengers	Sex, driving status, type of roads, no of passengers, the registration status of motorcycle, time of the day, day of the week, climate condition	Sex, driving status, type of roads, no of passengers, the registration status of motorcycle, climate conditions (only for passengers)	19
Ledesma [34]	2006	Mar del Plata, Argentina	Cross-sectional (observational)	451	Sex, type of motorcycle, climate condition, type of roads, time of the day	Sex, type of motorcycle, climate condition, type of roads	17
Ledesma [35]	2012	Mar del Plata, Argentina	Cross-sectional (observational)	2542 drivers and 664 passengers	Sex, driving status, type of motorcycle, climate condition, time of the day, day of the week, the registration status of the motorcycle, season	Sex, driving status, type of motorcycle, climate condition, time of the day, day of the week, the registration status of the motorcycle	18
Saeed [36]	Not mentioned	Karachi, Pakistan	Cross-sectional (survey)	400 female passengers	Education, opinions on helmet laws	Education	19
Roehler [37]	2011	Cambodia	Cross-sectional (questionnaire)	510 drivers, 410 passengers, and 99 proxied child passengers	Sex, driving status, reasons for using the helmet	Driving status	18
Grummon [38]	2010	Ohio, USA	Cross-sectional (questionnaire)	248 youth aged 9–15 years	Age, risky behavior, the parental reminder of helmet wearing, parental supervision, and perceived severity	The parental reminder of helmet-wearing, parental supervision, and perceived severity	20
Li [39]	2005	Shantou and Chaozhou, China	Cross-sectional (observational and questionnaire)	2325 interviews	Type of roads, time of the day, day of the week, KAP	Type of roads, time of the day, day of the week, KAP	17
Trejo [40]	2009	Mexico	Cross-sectional (observational)	26,046 drivers and 3971 passengers	Diving status, no of passengers, purpose of the trip, time of the day, day of the week, socioeconomic status of municipalities	Diving status, no of passengers, purpose of riding, socioeconomic status of municipalities	19
Grimm [41]	2011	Delhi, India	Two-stage cross-sectional (questionnaire)	1502	Age, sex, marital status, education, religion, helmet ownership, no of children, type of roads, type of motorcycle, history of crashes, length of trips, insurance possession	sex, education, religion, no of children, insurance possession, length of trips	15
Siviroj [5]	2007	Thailand	Cross-sectional (questionnaire)	18,998	Age, sex, engine size, time of the day, history of crashes, type of roads	Age, time of the day, history of crashes, type of roads	17
Kulanthayan [42]	1998	Kajang, Malaysia	Cross-sectional (observational and questionnaire)	500 (comparison between helmeted and non-helmeted or inappropriate helmeted riders	Age, sex, educational level, driving location, the length of the trips, driving experience	Age, sex, driving location, the length of the trips	20
Conrad [43]	1989	Indonesia	Cross-sectional (observational and questionnaire)	9242 drivers and 3541 passengers; 150 interviews	Sex, driving status, time of the day	Driving status, time of the day	19
Papadakaki [44]	Not mentioned	Greece	Cross-sectional (questionnaire)	405	Age, sex, education, time of the day, alcohol consumption, the purpose of the trip, history of crashes, attitudes, season	Sex, education, time of the day, alcohol consumption, the purpose of the trip, history of crashes, attitudes	20
Dandona [45]	2009	Hyderabad, India	Cross-sectional (questionnaire)	4183 drivers	Age, sex, education, type of motorcycle, motorcycle ownership, reasons for using helmets	Age, sex, education, type of motorcycle, motorcycle ownership, reasons for using helmets	16
Gkritza [46]	2000–2006	Iowa, USA	Cross-sectional (observational)	27,521	Climate conditions, type of roads, time of the day, month of the year, helmet sage by passenger/driver	Climate conditions, type of roads, time of the day, month of the year, helmet sage by passenger/driver	19
Zamani-Alavijeh [47]	2008–2009	Tehran, Iran	Cross-sectional (observational and questionnaire)	6010 drivers, 2215 passengers, and 31 (in-depth interviews)	Driving status, season, reasons for helmet use, history of crashes	-	15
Pileggi [48]	2004	Catanzaro, Italy	Cross-sectional (questionnaire)	898 youths (14–20 years of age)	Sex, alcohol consumption, a close friend who always uses helmets, driving status, history of crashes, performing sports activities, or riding over the speed limits	Sex, alcohol consumption, a close friend who always uses helmets, driving status	19
Jomnonkwao [49]	Not mentioned	Thailand	Cross-sectional (questionnaire)	401 urban and 400 rural drivers	Constitutes of HBM	Health motivation (i.e., importance of health; for urban areas), perceived severity (mortalities of not-wearing helmets), cue to action (advertisements), and perceived benefits (feeling safe; for rural areas)	21
Devagappanavar [50]	2017	Bangalore, India	Cross-sectional (observational)	1471 drivers and 920 passengers	Sex, driving status, driving location, time of the day, day of the week	Driving status, driving location, day of the week	11
Satiennam [51]	2012–2016	Khon Kaen Province, Thailand	Cross-sectional (observational, using an automatic helmet-use detection system)	42,821 drivers and 6,307 passengers	Age, driving status, number of passengers, time of the day, day of the week, type of motorcycle	Age (adults > children), driving status, number of passengers, time of the day, day of the week, type of motorcycle	18
Olewinski [52]	Not mentioned	USA	Cross-sectional (questionnaire)	125 athletes (scooter or moped drivers)	Reasons for helmet usage	The coach’s mandate has been the strongest predictor of helmet usage	19
Siebert [53]	2018	Nepal	Cross-sectional (observational and questionnaire)	1885 drivers and 663 passengers (observations); and 183 drivers and 36 passengers (interview)	Driving status, attitudes regarding fatalism, risk-personality, and helmet usefulness	Driving status, believing in fate as the cause of accidents, and presenting themselves as risk-taking persons have been higher among non-helmeted riders	20
Fletcher [54]	2016–2018	Jamaica	Cross-sectional (questionnaire)	155 hospital-admitted riders (139 drivers and 14 passengers)	Age, driving status, occupation status, type of motorcycle, license possession, insurance possession, riding training course	Age, driving status (only bivariate), type of motorcycle, license possession (only bivariate), insurance possession (only bivariate), riding training course	16
Fathollahi [55]	2016	Iran	Cross-sectional (questionnaire)	30,548	Age, sex, education, income, marital status, type of residence, alcohol consumption	Age, sex, education, income, marital status, type of residence (urban > rural)	18
Sankaran [56]	2015 and 2016	Mumbai, India	Cross-sectional (observational)	28,209 and 37,245	Age, sex, time of the day, day of the week, presence of passengers	Age, sex, time of the day, day of the week, presence of passengers	16
Merali [57]	2011–2016	Bangkok, Thailand	Cross-sectional (observational)	462 drivers	Type of roads, motorcycle use as taxi or non-taxi	Motorcycle use as taxi > non-taxi, type of roads (non-residential > residential)	19
Adewoye [58]	Not mentioned	Nigeria	Cross-sectional (questionnaire)	306 commercial drivers	Age, education, marital status, religion, the reason for using helmets	Age, education, marital status	20
Babazadeh [59]	2014–2016	Charuymaq, Iran	Cross-sectional (questionnaire)	150 rural drivers	Age, education, income, occupation, license possession, history of crashes, history of police fines	Education, income, history of police fines	18

*STROBE* Strengthening the Reporting of Observational Studies in Epidemiology, *No* number, *TPB* theory of planned behavior, *HBM* health belief model, *KAP* knowledge, attitude, and practice

**Table 2:** The significantly and positively related factors associated with helmet-wearing behavior

Variable	Positively associated with helmet usage^*^	Reference(s)
Age	More advanced age	[5, 12, 15, 18, 20, 26, 28, 29, 31, 42, 54–56, 58]
	Younger age	[45]
Sex	Women	[2, 17–21, 24, 26, 31, 33–35, 42, 44, 45, 56]
	Men	[12, 14, 27, 29, 41, 48, 55]
Education	Higher educational attainments	[19–21, 25, 26, 28, 30, 36, 41, 44, 45, 55, 58, 59]
Marital status	Married	[14, 15, 18, 19, 58]
	Unmarried	[2, 55]
Driving status	Drivers (compared to passengers)	[12, 17, 22, 24, 26, 28, 31, 33, 35, 37, 40, 43, 48, 50, 51, 53]
Number of passengers	Lower numbers	[5, 12, 17, 31, 33, 40, 51]
	Higher numbers	[56]
License possession	Licensed riders and motorcycles	[15, 19, 33, 35]
Helmet ownership	Helmet owners	[18, 19, 26]
Roads’ type	Highways	[21, 24, 39, 43, 46, 57]
	Central city roads	[5, 12, 17, 33, 34, 42, 50]
Timing of driving within the day	Mornings	[5, 17, 22, 24, 31, 39, 43, 46, 56]
Timing of driving within the week	Weekdays	[5, 17, 24, 31, 35, 39, 50, 51, 56]
Purpose of trip	Commercial (versus private) usage	[40, 57]
	From/towards home, work, or school/college	[26, 44]
Climate conditions	Sunny	[33]
	Rainy	[34, 35, 44, 46]
Length of trips	Longer distances	[28, 41, 42]
Frequency of trips	More frequent trips	[29]
	Less frequent trips	[15, 32]
Type of motorcycle	Larger engines	[24, 26, 31, 34, 45, 54]
	Smaller engines	[51]
History of previous crashes	Positive history	[28, 44]
	Negative history	[5, 21, 41]
Income	Higher income	[15, 20, 25, 27, 40, 55, 59]
Type of residence	Non-slum areas	[14]
	Urban areas	[55]

^*^ In studies with both univariable and multivariable analyses, only the multivariable analysis statistics have been considered significant

### Data analysis

The extracted quantitative data in this study are presented as frequencies (percent), mean, odds ratio (OR), 95% confidence interval (95%CI), and coefficient of determination (R^2^), if applicable [60]. Due to the considerable heterogeneity in the descriptions of included studies, we were not able to perform meta-analyses on the collected data. Hence, the meta-analysis-related statistical approaches do not apply to this study. We considered a P-value of less than 0.05 as statistically significant. For qualitative data, we reported their frequencies as the percentage and their associations with helmet usage as a coefficient of determination (R^2^). The quantitative and qualitative data calculations are conducted according to the established statistical guidelines for systematic reviews and meta-analyses [60]. To extract relevant statistics (mainly ORs and their CIs) from basic information provided by papers, we used Stata version 17 (StataCorp. 2021. Stata Statistical Software: Release 17. College Station, TX: StataCorp LLC.).

## Results

**Fig. 1:**
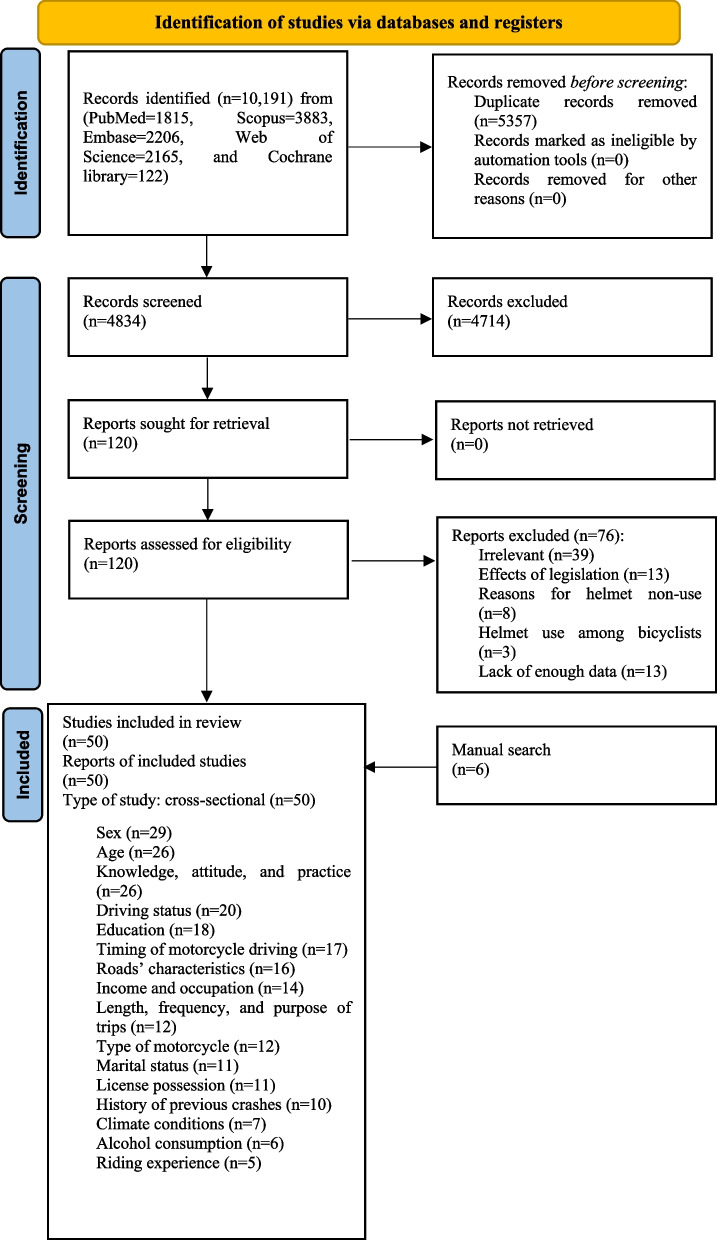
PRISMA 2020 flow diagram of the included studies in this article

Initial searches of the investigated databases produced a total of 10,191 titles. After reviewing the yielded titles from the mentioned databases and removing duplications, 4834 articles remained and underwent screening based on title/abstract evaluation. Thereafter, 120 titles were extracted for full-text screening. Eventually, 50 papers were determined as eligible to be included in this systematic review (Fig. 1). The median STROBE score was 18, with a mean of 17.94, and ranged between 15 to 21. None of the studies scored lower than the 50^th^ percentile, and ten studies (20%) fell within the 50^th^-75^th^ percentile (Supplementary Table 3). The characteristics of these articles are provided in Table 1. Supplementary Table 2 provides a more detailed summarization of each study’s findings. Each of the included articles was evaluated thoroughly for the factors that were positively associated with helmet usage.

### Demographic characteristics

#### Age

Regarding age, we found 26 studies that evaluated the effects of age on helmet usage behavior [2, 5, 12, 14, 15, 18–21, 24–31, 41, 42, 44, 45, 54–56, 58, 59]. In ten of these studies [2, 14, 19, 21, 24, 25, 27, 30, 44, 59], age was not a statistically significant determining factor for helmet use; however, there was considerable heterogeneity in the categorization of age groups. For instance, in Aidoo and colleagues’ study [19], using the unadjusted analysis, the helmet-wearing rate was significantly higher among those who were aged over 40 years (62.8%) compared to 25–40 (51.1%) and 16–24 (31.9%) age groups. However, the statistical significance of this difference disappeared in a multiple logistic regression model [19]. In Skalkidou and colleagues’ study [24], despite significantly higher helmet usage in older age groups, this did not remain significant in a multiple logistic regression model. In Ranney and colleagues’ report [21], they compared the mean ages of “always” helmet-user and “not-always” helmet-user riders (according to self-reports), for which there was no statistically significant difference (46.3 ± 12.7 versus 44.2 ± 13.3 years, *P* = 0.012). Similar outcomes have been demonstrated by Khan and colleagues’ study (32.5 ± 9.7 years for helmet users versus 30.9 ± 10.3 years for non-users; OR = 1.6, 95%CI = 0.0–3.9) [30]. In a cross-sectional study from an urban region in India [14], despite lower helmet usage rates among the elderly (over 60 years), no significant difference between the age groups was evident. However, owing to the restriction of the study participants to those who possessed a helmet, the findings of this study should be interpreted with caution [14]. In the Sreedharan [2], Ghasemzadeh [25], and Hernández [27] studies, there was no statistically significant association between helmet-wearing and age, which was dichotomized at values of 40, 18, and 59 years, respectively. In a study from Greece, the frequency of self-reported helmet usage was reported based on a score ranging between 0 and 5, and the correlation between age and this score was similarly not statistically significant [44].

Conversely, 15 studies have consistently identified associations between higher rates of helmet-wearing behavior and increased age of riders [5, 12, 15, 18, 20, 24, 26, 28, 29, 31, 42, 54–56, 58]. One important finding is that apart from the mentioned general trend, in three studies, the helmet usage rate exhibited a minor drop among the elderly [26, 29, 55]. Accordingly, in the first study, the helmet usage rate has been 25.4%, 36.5%, 36.2%, and 31.5% for those who have aged less than 24, between 25 and 39, between 40 and 54, and over 55 years, respectively [26]. The rates of the second study have been 48.1%, 53.1%, 66.1%, 72.8%, and 45.8% for drivers’ age groups of less than 18, 18–25, 26–40, 41–60, and over 60 years, respectively. The rates have been 26.8%, 23.8%, 26.5%, 38.4%, and 31.8% for the corresponding age groups of passengers [29]. In the third study, compared to the 45–54 years age group, individuals over 70 years have actually shown the lowest helmet compliance (OR = 0.4, 95%CI = 0.3–0.6; *P* < 0.001) [55]. It should also be noted that in Kumphong and colleagues’ study [31], due to its observational nature, riders have been classified into adults and children based on their appearance, but the trend of helmet-wearing has still been meaningful for both riders and passengers (67.3% versus 30.8% for children among the total cases, *P* < 0.001). Finally, in one study from India [45], the odds of helmet non-usage have been significantly higher among those aged 46 years and higher compared to 16–20 years old drivers, which is quite discordant with the descriptions of other studies.

#### Sex

In our search, we found 29 studies with reports about the importance of sex for helmet-wearing behavior [2, 5, 12, 14, 17–21, 24, 26–29, 31–35, 37, 41–45, 48, 50, 55, 56].

In eight studies [12, 14, 27, 29, 41, 48, 55], the helmet-wearing rate was significantly higher among men. In the Hernández report from Colombia, the helmet usage rate was 87.8% among men and 81.7% among women, which was statistically significant in multiple logistic regression models (adjusted OR = 2.0, 95%CI = 1.5–2.7). The authors have mentioned the unexpectedness of this finding but have not proposed any underpinning explanations [27]. In another study from Thailand [29], the self-reported “always-wearing” helmet behavior was 64.8% and 52.4% among male and female drivers, and 37.9% and 23.2% among male and female passengers, respectively. Moreover, significant negative associations were established between the female sex and helmet usage in multivariable ordered logistic models [29]. In Grimm and colleagues’ report from India, male passengers had a significantly higher chance of wearing helmets [41]. Mirkazemi and colleagues [14] reported that helmet possession was significantly higher among men (*P* < 0.001), and among those who own a helmet, again, men had significantly higher rates of its regular use (*P* < 0.001). Pileggi and colleagues reached similar results, as the odds of helmet usage were significantly higher among men in a logistic regression model [48]. In another large study from Iran [55], the age-adjusted OR of helmet compliance for women was 0.2 (0.2–0.2; *P* < 0.001) compared to men. In fact, rural females have exhibited the lowest probability of wearing helmets [55]. In Ackaah and colleagues’ report, despite significantly higher rates of helmet-wearing behavior among men in the total sample (OR = 1.92, 95%CI = 1.57–2.37), this was similar among both sexes when considering riders and passengers separately [12].

In three other studies (from Spain, Vietnam, and China), helmet usage was higher among men, but the differences did not meet the statistical significance level [20, 28, 33]. Of note, this description from Xuequn and colleagues’ study is only limited to drivers [33]. In five other studies from the USA [32], Ghana [12] (among riders and passengers, but not the total population), Thailand [5], Indonesia [43], and India [50], the helmet-wearing rate was similar for both sexes; however, it should be mentioned that the target population of Shults’s study was confined to 12–17 years old youths [32].

In 14 studies [2, 17, 20, 21, 24, 26, 31, 33–35, 42, 44, 45, 56], women constituted a statistically significantly greater proportion of helmet users, although marginally in some instances. It should be mentioned that in Xuequn and colleagues’ study [33], this finding was confined to passengers, and in the Kumphong study [31], the difference was significant in the total population.

#### Educational level

We found 18 studies [2, 14, 18–21, 25, 26, 28, 30, 36, 41, 42, 44, 45, 55, 58, 59] concerning the potential role of educational level in the decision of wearing helmets. Excluding four studies [2, 14, 18, 42], the remaining studies universally stated significantly higher helmet usage among those with more advanced educational attainments. In three studies [19, 26, 28], there were non-significant differences between those without formal education and with primary/secondary school degrees; however, the differences were statistically significant for those with higher levels of education. For instance, in Aidoo and colleagues’ report [19], the odds of wearing a helmet among those with a tertiary school degree was 35.29 (95%CI = 11.56–107.75, *P* < 0.001) times higher than that of those with no formal education. In Saeed and colleagues’ study [36], those with postsecondary or higher education showed significantly greater awareness regarding mandatory helmet laws (and not helmet-wearing behavior). There were also six studies that were from communities with different cultural and legal characteristics (USA [21], Spain [20], a rural area in Iran [25], a national survey from Iran [55], Pakistan [30], and Greece [44]), for which the corresponding P-values have been less than 0.01 for the first, less than 0.001 for the following four, and 0.032 for the last study.

In one study from India [2], the educational level between helmeted and non-helmeted riders was quite similar (*P* = 0.98), which might be attributed to the inaccurate categorization (below high school, high school/undergraduate, and graduate or above) and the limited number of those with a lesser than high school degree (20, compared with 138 and 151 for the other groups).

Finally, in a study from Ghana [18], the helmet usage rate was even numerically higher among those with no education (51.9% versus 46.3% for post-secondary/tertiary degrees), but the differences were not statistically significant.

#### Marital status

We identified 11 studies [2, 14, 15, 18–21, 30, 41, 55, 58] discussing associations between marital status and helmet usage. In one study from Ghana [18], married riders had significantly higher rates of commitment to helmet usage (OR = 0.44, 95%CI = 0.20–0.95, *P* < 0.001). In another study from India [14], helmet usage was significantly higher among married (48.6%) and separated (50%) riders compared to singles (43.8%, *P* = 0.007). In Aidoo and colleagues’ report, married subjects had significantly higher rates of helmet usage (OR = 2.3, 95%CI = 1.33–3.96, *P* = 0.003). A similar trend was also observed by Babio and colleagues [20] in a univariable model (OR = 1.41, 95%CI = 1.09–1.82); however, the multivariable logistic regression model did not corroborate that. In another three studies [21, 30, 41], despite a higher proportion of married subjects in helmet-user groups, the differences were not statistically significant.

Discordant with the abovementioned descriptions, in Sreedharan and colleagues’ report [2], the odds of unmarried riders regularly wearing helmets were 2.3 times higher than that of married ones (95%CI = 1.1–4.4, *P* < 0.05). Likewise, Fathollahi and colleagues [55] found that unmarried individuals have declared a higher commitment to helmet wearing (OR = 1.2; 95%CI = 1.0–1.4; *P* < 0.05) compared to married ones, yet this measure was 0.4 for widowed responders (0.3–0.6; *P* < 0.001). The authors of these studies did not provide any explanatory comments for their findings.

#### Income and occupational status

In our search results, 14 studies [14, 15, 18–20, 25, 27, 28, 30, 40, 41, 54, 55, 59] discussed the role of occupation and income in wearing helmets. In Aidoo and colleagues’ study [19], self-employed riders had the highest rate of helmet usage (52.4%), followed by employees (50.0%), students (29.4%), and unemployed individuals (16.0%). Despite significant differences in Χ^2^ statistics (*P* < 0.001), the adopted multiple logistic regression model has shown that none of them are statistically different compared to students. As another example, Ghasemzadeh and colleagues [25] did not show a difference in helmet usage rates between various occupational conditions. Accordingly, Hung and colleagues [28] did not find significant differences between professional or non-professional jobs and farming regarding helmet usage. Similarly, helmet usage was not different among various classes of monthly and average income per head [28]. In Khan and colleagues’ report [30], despite a higher income among helmet users on univariable analysis, the difference was not significant in multivariable analysis. Again, Mirkazemi and colleagues [14] found that the proportion of riders who owned helmets was associated with their socioeconomic status (*P* = 0.005); however, among the helmet owners, the correlation between socioeconomic status and helmet usage was not significant (*P* = 0.091). Of note, this study also found significant correlations between regular helmet usage with belonging to a nuclear or single-parent family and residence in non-slum areas [14]. Likewise, in another study from Iran, residents of urban areas showed higher helmet-wearing commitment (OR for residency in rural areas = 0.6, 95%CI = 0.6–0.8) [55]. However, the type of residence (urban versus rural, and living within or outside the metropolitan statistical area) was not a significant determinant of helmet usage in two other papers [28, 32].

On the other hand, in Babio and colleagues’ report [20], those with an average family income of 7,300–8,800 euros had an OR of 1.45 for wearing helmets, compared with their counterparts with less than 7,300 euros of income (95%CI = 1.01–2.08, *P* < 0.05). The OR for wearing a helmet was also 2.35 for the group with at least 8,800 euros of income (95%CI = 1.55–3.56, *P* < 0.01). Extending the socioeconomic status to larger dimensions, in Hernández and colleagues’ report [27], the strongest predictor of helmet usage was the city that the respondents lived in (82.4% for Valledupar versus 98.1% for Ibagué), and they proposed lower social and economic development as the main culprits of lower helmet usage in Valledupar. Similarly, Trejo and colleagues [40] found higher helmet usage in municipalities with higher socioeconomic levels.

#### Religion

Regarding religious beliefs, we found five relevant articles [2, 36, 41, 47, 58]; however, the descriptions are quite heterogeneous, and robust conclusions cannot be made. As such, in Sreedharan and colleagues’ study [2], despite a higher rate of regular helmet usage among Christians (34.6%), compared no Muslims (25.9%) and Hindus (25%), this was not statistically significant. Likewise, in Adewoye and colleagues’ report from Nigeria [58], the differences between Muslims (30%), Christians (22.5%), and traditional worshippers (28.6%) were not significant. However, this might be biased by the larger number of included Christians (262) compared to Muslims [45] and traditional worshippers [20]. Again, in Grimm and Treibich’s report [41], religion was not a determinant of helmet-wearing behavior in passengers, and among drivers, this was only limited to lower rates in Sikhs compared to Hindus.

In another study aimed to assess the perception regarding helmet usage in the woman [36], despite the general agreement on the considerable effects of religious beliefs on attitudes and behavior, no differences were found between Muslims, Hindus, and Christians regarding their awareness of helmet laws (Christians, 36.3%; Muslims, 32.8%; and Hindus, 30.7%) and their positive opinion regarding helmet usage as a passenger (Christians, 100%; Muslims, 81.8%; and Hindus, 69.2%). Moreover, Religion was not cited by women as the main reason for helmet-abiding behavior [36]. Finally, in a report from Iran, the authors have only mentioned that as riders are breadwinners, they are compelled to stay safe according to religious pieces of advice [47].

### Riding-related factors

#### Driving status

We found 20 studies [5, 12, 17, 22, 24, 26, 28, 31, 33, 35, 37, 40, 43, 47, 48, 50, 51, 53, 54, 56] comparing helmet usage rates of drivers and passengers and the impact of the presence of passengers on the helmet-wearing behavior of drivers. In a report from Cambodia, drivers have had up to 10 times higher helmet usage rates than passengers [22], and a similar finding was also described by Skalkidou and colleagues’ report from Greece [24]. Ackaah and colleagues [12] also documented a total helmet usage rate of 34.2% for drivers and only 1.9% for passengers (*P* < 0.001), and hence, drivers have had an 18 times higher chance of wearing helmets. Likewise, in Akaateba and colleagues’ report [17], 45.8% of drivers and only 3.7% of passengers were helmet users. Wadhwaniya and colleagues [26] found that the prevalence of helmet usage was 34.8% among Indian drivers and 20.6% among passengers, leading to an OR of 1.8 (95%CI = 1.1–3.1, *P* < 0.05). In the multivariable analysis of Hung and colleagues’ paper [28], the odds of wearing helmets was 3.71 for drivers compared to passengers (95%CI = 1.69–8.14, *P* = 0.0011).

The findings of Xuequn and colleagues’ report [33] showed that 72.6% of drivers versus 34.1% of passengers were helmet users. They further tried to assess the associations between the number of passengers and helmet-wearing rate. Compared with drivers with no passengers, those with one or at least two passengers have had significantly lower probabilities of wearing helmets (OR for not wearing helmets = 1.27, 95%CI = 1.16–1.38, *P* < 0.001; and OR for not wearing helmets = 1.41, 95%CI = 1.13–1.75, *P* = 0.002, respectively). Ackaah and colleagues [12] reached similar results, as the helmet usage rate of drivers with at least one passenger was 27.4%, compared with 37.3% for drivers without passengers (*P* < 0.001), a finding that was also documented by Akaateba and colleagues [17]. In line with these descriptions, Trejo and colleagues [40] also found lower helmet usage rates among passengers compared to drivers (OR = 0.15, 95%CI = 0.14–0.17), and among drivers with at least one passenger, compared to solo drivers (OR = 0.49; 95%CI = 0.45–0.54). Likewise, in another study from Thailand, having at least one passenger was one of the strongest predictors of helmet non-use by drivers [5]. A similar finding was also demonstrated for passengers without and with at least one additional passenger (OR = 12.79, 95%CI = 8.98–18.21, *P* < 0.001), in which the drivers without passengers exhibited a higher probability of wearing helmets. The findings of Kumphong and colleagues’ study [31] regarding the impact of the number of passengers on helmet-wearing were also in accordance with other studies (helmet usage rate of 75.1% for riders without passengers, compared to 53.9% for riders with at least one passenger; OR = 1.55, 95%CI 1.46–1.66, *P* < 0.001). Added to these, Ledesma and colleagues [35] have reported a helmet usage rate of 69.8% among drivers and 43.4% among passengers. In addition, they have found that helmet-wearing by the driver was positively associated with this behavior among passengers. Finally, the findings of Roehler and colleagues (50% among drivers versus 14% among passengers, *P* < 0.001) [37] further corroborate the aforementioned results.

Despite the abovementioned findings, in a large observational study from India [56], drivers with a passenger were significantly more committed to helmet-wearing than those without passengers.

#### Length, frequency, and purpose of trips

We found 12 studies [15, 19, 21, 26, 28, 29, 32, 40–42, 44, 57] concerning the putative role of the trip’s duration, purpose, and frequency on helmet-wearing behavior. In Aidoo and colleagues’ observation [19], helmet usage was more common among those who travel longer distances (55.9% for longer than 2 km [km] versus 35.95% for less than 2 km trips, *P* < 0.001) and ride motorcycles more frequently (51.2% for every day, 44.6% for 3–6 times per week, and 23.3% for less than three times per week users, *P* = 0.002), but none of them were statistically significant in a multivariable logistic regression model. In another study from India, longer trips showed higher rates of helmet-wearing behavior [41]. The descriptions of Hung and colleagues [28] corroborate the aforementioned findings. In fact, the distance of trips was the strongest predictor of helmet-wearing behavior in this study. Those with trip lengths of more than 10 km had an OR of 23.29 for wearing helmets, compared to those with trips less than 2 km (95%CI = 8.05–67.40, *P* < 0.0001). Although the helmet usage rate was higher for riders with more frequent trips, the differences were not significant [28]. In Ranney and colleagues’ report [21], 24.4% of “not-always” helmet users, versus only 2.4% of “always” helmet users stated that they need safety helmets only for long trips (*P* < 0.001). The findings of Jiwattanakulpaisarn and colleagues [29] regarding positive associations between the driving frequency and wearing helmets are in concordance with previous descriptions.

Notably, the study of Shults and colleagues [32] led to an opposite conclusion, as the estimated rate of helmet usage was 81% among those with one trip per week, 68% for two to five trips per week, and less than 20% for at least six trips per week. Of note, Adnan and Gazder [15] found that the distance of trips, and also mean fuel consumption, does not affect the decision to wear helmets; nevertheless, less frequent daily trips have been a significant contributor to the post-campaign population’s helmet usage, and have been a key element for this behavior in a non-parametric classification and regression tree (CART) model (discussed later) [15].

Regarding the intention of trips, a study from India [26] found that college, school, or work-related trips showed higher rates of helmet usage. Likewise, in Papadakaki and colleagues’ study [44], self-reported helmet-wearing was higher for trips to home, work, or school. In a study from Mexico, helmet usage was more common among riders of commercial motorcycles (i.e., food delivery) than the riders of private motorcycles (OR = 1.76; 95%CI = 1.59–1.96) [40]. Lastly, a study found significantly higher helmet usage among drivers of taxi motorcycles compared to those of non-taxi applications [57].

#### License possession

In our search results, we found 11 studies [15, 18, 19, 21, 24, 25, 28, 33, 35, 54, 59] that assessed whether the possession of a driving license affects helmet-wearing behavior. In Aidoo and colleagues’ study from Ghana [19], license possession was significantly correlated with helmet wearing (OR = 3.75, 95%CI = 1.90–7.42, P < 0.001). Accordingly, Xuequn and colleagues [33] found the prevalence of helmet-wearing behavior to be 72.9% and 22% among drivers of licensed and unlicensed motorcycles (indicated by the detection of registration plates on motorcycles). The corresponding rates for passengers were 34.2% and 0.8%, respectively. These observations were significant among both drivers (OR for helmet non-usage = 12.26, 95%CI = 7.21–20.84, *P* < 0.0001) and passengers (OR for helmet non-usage = 8.57, 95%CI = 2.22–33.08, *P* = 0.002), making it (i.e., license possession) one of the strongest predictors of helmet usage/non-usage among both drivers and passengers. Again, Ledesma and colleagues [35] concluded similar results, as helmet usage was significantly more common among drivers (OR = 0.42, 95%CI = 0.31–0.56, *P* < 0.001) and passengers (OR = 0.56, 95%CI = 0.34–0.93, *P* = 0.024) of licensed motorcycles.

However, other studies reported different results. For instance, Ranney and colleagues observed high rates of license possession among both “always” (98.2%) and “not-always” helmet wearers (97.6%), which might stem from the fact that the target population of this study was newly graduated U.S. riders from motorcycle training courses [21]. On the other hand, in Ghasemzadeh and colleagues’ study from a rural area [25], the proportion of license holders was just 17.5% and 15.7% among helmet users and non-users, respectively. In another report from Greece [24], the prevalence of unlicensed drivers and passengers was 4% and 82%, respectively, and the difference between helmet-using and non-using groups was higher only for passengers (*P* = not mentioned). In Hung and colleagues’ report [28], the positive impact of license possession on helmet usage was significant in the univariate (OR = 2.73, 95%CI = 1.34–5.73) but not in the multivariate model. Likewise, in another study [18], the license holders have had a higher rate of helmet usage (65.4% versus 41.6%), but the difference has been significant only in the unadjusted model (adjusted OR = 1.02, 95%CI = 0.47–2.23).

#### Type of motorcycle

Among our included titles, 12 [5, 21, 24, 26, 28, 31, 34, 35, 41, 45, 51, 54] discussed the possible interactions between the type of driven motorcycle (different classes and/or engine capacities) and helmet usage. In Skalkidou and colleagues’ study [24], there was a significant relationship between the engine size category and helmet usage (14.3% for engines less than 50 cc, 12.6% for 51-200 cc, 31.8% for 201-400 cc, and 32.5% for greater than 400 cc engines), in which an increase in the category of the engine had an OR of 1.52 for helmet usage (95%CI = 1.30–1.75, *P* = 0.0001). Wadhwaniya and colleagues [26] reached similar conclusions (adjusted OR for helmet usage = 1.6 for engines > 100 cc compared to engines < 100 cc, 95%CI = 1.0–2.4, *P* < 0.05). However, it should be mentioned that only 2.5% of respondents had a motorcycle with an engine smaller than 100 cc. Likewise, in Kumphong and colleagues’ report [31], riding a motorcycle with an engine size of more than 125 cc was associated with increased helmet usage among the total population (OR = 1.91, 95%CI = 1.71–2.15, *P* < 0.001) and drivers (OR = 1.98, 95%CI = 1.745–2.25, *P* < 0.001), but not passengers. In one of Ledesma and colleagues’ studies [34], they reported significantly lower helmet usage among drivers of scooters and Cross/enduros compared to drivers of motorcycles with 250 cc engines. Similar descriptions are also recorded by Dandona and colleagues [45], as the odds of not wearing a helmet were significantly higher among drivers of mopeds and scooters compared to motorcycles. In a study from Thailand [51], drivers of motorcycles with an engine size of less than 125 cc wore helmets at a rate of 2.1 times that of others. Interestingly, this difference disappeared after the establishment of mandatory helmet-wearing enforcement using closed-circuit television (CCTV) cameras.

However, in another report by Ledesma and colleagues [35], no significant differences in terms of helmet-wearing were observed among riders of different types of motorcycles (moped, street standard, custom/sport, and off-road). Likewise, three other studies did not observe important differences in helmet usage rates between the different types [21] and engine sizes [5, 41] of motorcycles.

#### History of previous crashes

Ten studies [5, 19, 21, 25, 28, 41, 44, 47, 48, 59] evaluated the impact of a positive history of motorcycle crashes on the decision to wear or not safety helmets. In Aidoo and colleagues’ study [19], despite meaningfully higher rates among those with a positive history, there was no significant association between these two variables in a multivariable logistic regression model. Hung and colleagues [28] reported an OR of 2.01 for wearing helmets for those with positive crash history, compared with their negative counterparts (95%CI = 1.09–3.71, *P* = 0.03). A similar trend was documented by Papadakaki and colleagues [44]. In a qualitative study, the experience of previous crashes was a positive influence on helmet usage [47].

On the other hand, in Ranney and colleagues’ article [21], 62.2% of “not-always” helmet wearers reported a previous motorcycle accident, compared to 25.3% of “always” helmet wearers (*P* = 0.01). Ghasemzadeh and colleagues [25] observed a similar trend (positive history in 13.8% of helmeted versus 18.6% of non-helmeted drivers), but the difference was not significant. Similar findings were also documented by Grimm and colleagues (among both drivers and passengers) [41] and Siviroj and colleagues’ studies [5].

#### Riding experience

There were five studies [15, 19, 24, 28, 42] that surveyed their target populations about their driving experience and its possible association with helmet-wearing behavior. In Aidoo and colleagues’ report [19], 30.2% of drivers with less than two years of experience were helmet users, while this was 46.5% for those with 2–5 years, and 50.8% for those with more than five years of experience. However, the significance of these differences was limited to the Χ^2^ test (and not the multivariable logistic regression model). The results of two other studies [24, 28] were the same as above, i.e., despite statistically significantly higher helmet usage among drivers with more experience in univariable analyses; these differences lost their significance in multivariable logistic regression models. In another study [15], helmet usage was significantly correlated with higher mean years after receiving the driving certificate before and after the implementation of a helmet enforcement campaign. However, this variable does not necessarily corroborate higher experience and should be interpreted with caution.

#### Helmet and motorcycle ownership

We found five papers [15, 18, 19, 22, 26] delineating possible correlations between helmet ownership and its use. In one of these studies from Ghana [18], helmet owners demonstrated higher helmet usage rates than non-owners (53.7% versus 24.3%, adjusted OR = 2.56, 95%CI = 1.24–5.27, *P* < 0.001). Likewise, Aidoo and colleagues [19] found helmet ownership to be a principal predictor of helmet usage; in fact, it was second only to the educational level in terms of the effect size (OR = 2.1, 95%CI = 8.3–48.65, *P* < 0.001). Of note, this variable was also the most powerful predictor of helmet wearing in Wadhwaniya and colleagues [26] report (adjusted OR = 40.4, 95%CI = 20.8–78.5, *P* < 0.001). However, in Bacahni and colleagues’ study [22], helmet ownership was not significantly different between helmet users and non-users. In Adnan and Gazder’s study, previous experience of helmet stealing was associated with its higher usage in both pre- and post-campaign surveys; however, the interpretation of this finding needs further research, as the ownership status of helmets is not discussed in this paper [15].

Concerning motorcycle ownership, in one study, it was not significantly associated with observed (OR = 1.3, 95%CI = 0.8–2.2) and self-reported (OR = 1.6, 95%CI = 1.0–2.5) helmet usage after adjusting for co-variates [26]. However, another study found this to be a significant determinant for this behavior (OR of borrowed versus owned vehicle for no/occasional helmet usage = 7.89, 95%CI = 3.39–18.40) [45].

#### Alcohol consumption

Six studies [2, 21, 28, 44, 48, 55] surveyed motorcycle riders about alcohol consumption. In the first study, those who have not been affected by alcohol during the ride had a greater chance of wearing helmets [2]. In Ranney and colleagues’ study, 100% of “always” helmet users versus 96% of “not-always” helmet users believed that riders should not drink alcohol before traveling on a motorcycle (*P* < 0.001) [21]. In another study from Greece [44], the consumption of high-concentrated alcohol (versus low alcohol-capacity beverages) was inversely associated with self-reported helmet usage. In the third study; however, there were no significant differences between those with and without alcohol consumption within one hour before their trip [28].

However, in Pileggi and colleagues’ study [48], those who have not been current alcohol drinkers had lower odds of using helmets (OR = 0.45, 95%CI = 0.25–0.82). Likewise, in Fathollahi and colleagues’ report [55], those with a positive history of alcohol consumption had a marginally higher, although non-significant, helmet compliance (OR = 1.1, 95%CI = 0.6–1.9).

### Environmental factors

#### Roads characteristics

Sixteen papers [5, 12, 17, 21, 24, 27, 28, 33, 34, 39, 41–43, 46, 50, 57] were identified that evaluated the possible predictive role of the type of driven roads for wearing helmets. In two studies from Ghana [12, 17], the authors observed higher helmet usage rates among drivers of the central business district (CBD) than in other regions (36.5% versus 32.1%, *P* = 0.0096; and 48.9% versus 42.3%, OR = 1.23, 95%CI = 1.14–1.34, *P* < 0.001, respectively). Likewise, a study from Malaysia observed a higher proportion of riders with correct helmet usage (compared to non-helmeted riders and those with inappropriate usage) in the city regions [42]. Accordingly, Ledesma and colleagues found higher helmet usage rates in the city central area compared with macro-center (although non-significant) and peripheral areas [34]. In another study from China [33], a similar observation was made, as 96.8% of city roads’ riders were helmet-wearers, compared with 74.6% for provincial roads, 65% for country roads, and 63% for national roads (the latter three types are considered to as rural roads). Interestingly, the highest ORs for not-using helmets were reported for the national roads’ drivers and passengers, compared with those of city roads (OR for drivers = 16.93, 95%CI = 13.44–21.32, *P* < 0.0001; OR for passengers = 30.8, 95%CI = 22.28–42.56, *P* < 0.001), making road type the strongest predictor of helmet-wearing behavior in this study [33]. Last but not least, according to Siviroj and colleagues’ cross-sectional report [5], the proportion of non-helmeted drivers was higher on trips on highways and roads out of town compared to the main roads.

However, different descriptions are reported in a study from the USA [46], in which higher helmet commitment was observed on secondary and primary roads in comparison with city roads (which have lower speed limits). In another one of these studies from Greece [24], the helmet usage rate was 80.8% among riders on highways, while it has been only 16.3% and 9.7% for the main road and suburban road riders (*P* < 0.001). Besides, a multivariable logistic regression model demonstrated that the highest OR for helmet usage was for highway riders, compared with those of main roads (OR = 6.25, 95%CI = 4.35–9.09, *P* < 0.001) [24].

Of note, in Hung and colleagues’ report [28], the OR for helmet usage was marginally higher for compulsory roads (OR = 2.05, 95%CI = 1.11–3.79, *P* = 0.02). This more strict law enforcement was also proposed as the putative reason for the higher helmet commitment of drivers riding on central city roads [50].

Finally, in Hernández and colleagues’ observation [27], despite significantly higher rates of helmet usage among drivers of three-lane roads (98.1%) compared to two-lane roads (84.6%, *P* < 0.001), the difference was not significant in multivariable analysis.

#### Timing of motorcycle driving

We identified 17 studies [5, 15, 17, 22, 24, 31, 33–35, 39, 40, 43, 44, 46, 50, 51, 56] assessing the importance of driving on different days of the week and/or different hours of the day for the commitment to using helmets. Bachani and colleagues [22] found that the helmet usage rate is 43% in the daytime and only 25% at night and the differences were statistically significant for both drivers and passengers. A similar observation was reported by Skalkidou and colleagues [24] (23.5% at daytime versus 13.1% at night, *P* < 0.001); however, they were not able to find a significant difference for the different days of the week (21.3% for weekdays versus 17% for weekends, *P* = 0.082) despite significance at the multiple logistic regression model. Akaateba and colleagues [17] documented significantly higher usage rates in the mornings (49.8% versus 44.4% in the afternoon and 42.5% in the evening, adjusted OR for morning versus evening = 1.25, 95%CI = 1.15–1.39, *P* < 0.001) and at the weekdays (49.2%, versus 38.5% for weekends, adjusted OR = 1.57, 95%CI = 1.45–1.70, *P* < 0.001). In Kumphong and colleagues’ article [31], among the total studied individuals, the helmet usage rate was 79.7% in the mornings, 74% in the afternoons, and only 47.2% in the evenings (OR for morning = 5, 95%CI = 4.74–5.37, *P* < 0.001; OR for afternoon = 3.8, 95%CI = 3.57–4.06, *P* < 0.001). The corresponding rates for weekdays and weekends were 68.5% and 64.1%, respectively, but the difference was not significant. Likewise, Siviroj and colleagues [5] documented increased chances of helmet non-use with the advancement of daytime (compared to the 7:00–9:00 time interval). Separating drivers and passengers, timing remained significant for both, while the day of the week became significant for drivers only [31]. Ledesma and colleagues [35] found that the only significant difference for these variables belonged to the higher helmet usage among drivers on weekdays (*P* < 0.005).

However, in Trejo and colleagues' report from Mexico [40], there were no significant differences between the days of the week. In addition, except for the lower helmet usage rate for the 10:30–14:00 time interval (OR = 0.61, 95%CI = 0.46–0.81), there were no differences between the afternoon and evening times with the 7:00–10:30 interval [40]. In Conrad and colleagues’ observation [43], despite more prevalent helmet usage by drivers and passengers in the morning hours (compared to afternoon and evenings), the differences were not statistically significant for all but one location. In Papadakaki and colleagues’ report [44], self-reported helmet usage was highest during the 14:00–22:00 time period, followed by 6:00–14:00 and 22:00–6:00 intervals.

Finally, the descriptions of Xuequn and colleagues’ paper [33] somehow differ from the others, as the helmet usage rate for both drivers and passengers was higher in the evenings (65.8%, 72.1%, and 89.1% for drivers at 7, 9, and 17–19 and 28.4%, 33.4%, and 60.3% for passengers at the corresponding times). However, despite these differences and also higher usage rates on weekdays, none of them were statistically significant predictors of helmet usage in multivariable logistic regression models.

#### Climate conditions

In our search results, seven studies [27, 33–35, 44, 46, 47] investigated the predictive value of climate conditions on helmet-wearing behavior.

In a Colombian study [27], helmet usage was higher on dry days (87.3% versus 80.9% for days with light rainfall); however, the difference was not significant in a multivariate model. Similarly, in Xuequn and colleagues’ paper [33], the helmet usage rate on sunny, cloudy, and rainy days was 73.4%, 71.7%, and 67.2% for drivers; and 36.9%, 31.5%, and 18.4% for passengers, respectively. Nevertheless, in a multivariable logistic regression model, only cloudy days (compared with sunny days) were a significant predictor of non-helmet usage by passengers (OR = 1.3, 95%CI = 1.09–1.55, *P* = 0.004).

The converse was the case for the other two [34, 35] studies (both from Argentina), as helmet usage was higher under bad climate conditions. In the first study [34], the odds of helmet usage on rainy days were 8.1 times more than that of good weather conditions (95%CI = 3.98–16.40, *P* < 0.001), which made it the strongest predictor of their model. In the second report [35], a similar trend was observed, as helmet usage was highest on rainy days, followed by cloudy and sunny days. However, in a multivariable logistic regression model, the statistical significance of this variable was limited to drivers only [35]. Likewise, Gkritza [46] reported a significant difference in helmet usage by drivers and passengers between sunny and cloudy or rainy days in favor of rainy days. Moreover, helmet usage by drivers was significantly higher in April compared to August, which might be attributable to the higher chance of rain during April and the improved self-confidence in skills during the time in each year [46]. According to a report from Iran [47], helmet-wearing behavior was more common among drivers during winter (compared to spring), although no statistical data was reported. Papadakaki and colleagues found that self-reported helmet usage was higher under bad climate conditions and was lower during spring compared to other seasons (although the differences were non-significant) [44]. On the other hand, another study [35] found similar helmet usage rates among both drivers and passengers during spring versus winter.

### Knowledge, attitude, and practice

The attitude of motorcycle riders regarding safety helmets and their reasons for using them was a matter of interest for 26 studies [5, 16, 18, 21–25, 28–31, 37–39, 43–49, 52, 53, 58, 59]. In Ranney and colleagues’ study [21], the most powerful correlate of not always wearing helmets was the attitude that helmets cannot be protective. In addition, there were significant differences regarding norms (e.g., “all of my friends who ride motorcycles wear helmets”, “I would only wear a helmet if the law made me”, and learning motorcycle riding from a professional course) and behaviors (such as always wearing seat belts and protective clothing and never speeding on a motorcycle) between the two groups (*P* < 0.001 for all variables) [21].

Ghasemzadeh and colleagues [25] found that among the constructs of the theory of planned behavior (TPB), attitudes (*r* = 0.667, *P* < 0.05), subjective norms (*r* = 0.761, *P* < 0.05), and perceived behavioral controls (r = 0.606, *P* < 0.05) were correlated with helmet usage. Similarly, Hung and colleagues [28] performed qualitative research regarding attitudes and beliefs about helmet usage. In their study, most drivers, regardless of their helmet usage status, stated that helmets are protective during accidents. Based on this result, the authors have concluded that public education programs are not of substantial effectiveness unless strict laws and observations come into action. In addition, both groups strongly disagreed with the statement that skilled drivers do not need helmets; however, a significantly higher proportion of non-helmet users agreed that helmets are not needed for short trips and slow and careful driving styles. This study also found that a substantial proportion of either helmeted or non-helmeted motorcycle drivers believe that helmets interfere with their auditory and visual functions, implicating the importance of helmet design and the unmet need to enhance it [28]. Likewise, another study documented that despite the general agreement regarding the protective functions of helmets, most will not use them, and even among the users, it is more because of fear of police fines [47]. The significance of the positive associations between beliefs and attitudes toward helmet usage was also stated by Akaateba and colleagues’ report [18].

To further evaluate the possible contribution of behavioral factors, one study delineated the associations between components of the TPB and health belief model (HBM) and the choice of wearing helmets [16]. In this study, perceived behavioral control (i.e., self-assessed ability to a certain behavior) was a significant predictor of the intention to use helmets (R^2^ = 0.47, *P* < 0.001). In addition, perceived behavioral control, as well as perceived barriers, self-efficacy, and cues to action (components of HBM), were identified as predictors of helmet usage behavior (R^2^ = 0.35, *P* < 0.001). These findings implicate the prominent roles of social factors, including encouragement by family members and friends, empowerment of self-confidence, and health education campaigns [16].

In line with the importance of friends’ and families’ influence, three other studies [47, 48, 52] also mentioned the importance of these factors on helmet-wearing behavior; such as having an “always helmet”-wearer close friend [48], the encouragement by family members and friends, and when mandated by a coach [52], have all been facilitators of helmet usage, and conversely, negative labels used by a friend have been a barrier [47]. Similarly, in Papadakaki and colleagues’ study, self-reported helmet usage was higher among those who declared they adopt the good practices of their family members and friends [44]. This might also be attributed to the description of Gkritza [46], in which helmet usage by passengers had a positive impact on performing this behavior by drivers. In a study regarding determinants of helmet-wearing behavior among youth, parental reminders, their supervision, and also youth self-efficacy had significant impacts on this choice [38].

Regarding the reasons behind using helmets, a study from Cambodia [22] documented the most commonly stated reasons to be their protective functions against crash-related mortalities (86%), the legal duty of wearing them (25.3%), and police fines (21.3%). Similarly, Roehler and colleagues [37] found that 96% of drivers and 98% of passengers who regularly use helmets cite the helmet’s lifesaving potential as the main reason for using it. In Dandona and colleagues’ study [45], 69.5% of “always-helmet” users stated its safety benefits as their reason for using it, and 26.7% stated their reason as protection from pollution. In Li and colleagues' study [39], helmeted drivers declared that they wear helmets to protect themselves from injuries (74.1%) and to cope with the police (20%). In Akaateba and colleagues' article [18], the reasons were to prevent head injuries (82%), respect law enforcement (59%), establish protection against dust and wind (47%), and avoid police fines (30%). More importantly, 97% of non-helmet users in this study also agreed that helmets are protective against head injuries and that they are aware of law enforcement (81%) [18]. Khan and colleagues [30] reported the most common reasons for helmet usage as the following: its protective role against injuries (78%), its protective role against dust (50%), and a positive history of previous falls (35%). Furthermore, they found a significant difference between helmet users and non-users regarding their belief about the protective role of helmets, but this was not found for their awareness of relevant traffic laws [30].

In another study by Siviroj and colleagues [5], the riders who had a history of previous crashes, and more notably, being previously caught by police due to helmet non-use, were more prone to helmet non-use behavior, implicating the pattern of “persistent high-risk behavior” among them. Likewise, Kumphong and colleagues [31] noticed the red-light running behavior as a significant negative determinant of helmet usage (OR = 0.59, 95%CI = 0.52–0.65, *P* < 0.001). Conversely, Pileggi and colleagues did not find significant associations between always riding over the legal speed limits and self-reported regular helmet usage [48].

### Studies with special aims

A four-year cross-sectional observational study of 62,039 passengers aged 12 years and lower (measured subjectively) [13] found the helmet usage rate to be as low as 2.1%. The analyses showed that helmet usage by child passengers was affected by the helmet wearing by the driver (3.5% versus 0.5% for non-wearing helmet drivers; OR = 6.2), the number of child passengers (2.4% for one child versus 1.3% for two or more children), time of the day (highest rate for noon at 2.6%, and lowest rate for 7 pm at 1.6%), the day of the week (highest rate on Sunday and Monday at 2.3% and the lowest rate on Wednesday at 1.9%), and the province of origin. According to the results of a multivariable logistic regression model, the helmet usage by the driver had the highest OR for helmet usage by children, emphasizing the importance of parental role modeling, their attitudes, and perceived behavioral controls. As discussed earlier, the differences in the time and the day of the week can be justified by the more prominent presence of law enforcement on Sundays, 12 pm, and 5 pm, compared to Fridays, Saturdays, and 9 am. In line with that, provinces with higher helmet usage are more urban, and Phnom Penh, with the highest helmet usage rate, is the capital [13].

In an extensive analysis from Pakistan [15], the authors aimed to investigate the determining factors of helmet-wearing among two different populations, before and three weeks after the conduction of an enforcement campaign. In both pre- and post-campaign groups, the holding of a driving license, higher intervals since license acquisition (in terms of years), previous experience of helmet theft, and experience of hospitalization (at least three days) following motorcycle crashes were found to be significant determinants of helmet usage. Among the pre-campaign group, couples (compared to singles), those with higher age, and those with higher monthly income had higher rates of helmet-wearing commitment. In contrast, in the post-campaign group, a lower mean number of daily trips appeared to significantly affect helmet usage [15]. In none of these groups, the length of trips, time of the day in which trips have been made, and educational level (compared with the student’s T-test within each level of education) were significant determinants of helmet usage. Moreover, the authors performed a CART model, in which the possession of a license appeared as the most important determinant of helmet usage, followed by the number of daily trips and the age of riders. Last but not least, this study found no considerable effects from the enforcement campaign [15].

## Discussion

In this systematic review, we aimed to determine factors that are positively associated with helmet usage behavior among motorcyclists. Various demographic and environmental factors were identified in this study (Table 2). Among all, the driving status was almost the only one that was universally stated by published studies as a contributor to helmet usage. Why passengers have lower helmet usage rates is not systematically discussed; however, owing to the descriptions that the number of passengers negatively correlates with helmet-wearing by drivers and passengers [33], it seems that the lack of enough available helmets may play a role [17]. Moreover, a lack of law enforcement for passenger helmet usage might also be conducive [12, 22, 33]. As an example, in a report from Nepal, the helmet usage rate was 98.7% among drivers and surprisingly only 0.8% among passengers, which was attributed to the absence of fines for unhelmeted passengers, despite mandatory laws [53]. It should also be mentioned that motorcycles serve as taxis in some Eastern-Asian countries (moto-taxi). Thus, passengers of such motorcycles seem to deny using helmets due to hygienic excuses, the short duration of their trips, or even more principally, the lack of helmets by drivers [17, 27]. It seems reasonable, therefore, to put more emphasis on the fact that motorcycle passengers are just like their drivers, and the law should also face them equally.

Regarding sex, we found heterogeneity in our findings. It is evidenced that women are usually more risk-averse than men [17, 61] and are more concerned about the probability of the occurrence of a certain accident [62]. In our findings, in seven studies, men had significantly higher rates of helmet usage. We believe that to deal with the missing point in delineating these paradoxes one should focus on the social and cultural disparities. As an illustrating example, in a study from Pakistan [36], 98.5% of female motorcycle riders stated that they would wear a helmet if they were male. They further referred to the rarity of helmeted female riders and that this will draw negative attention if they wear a helmet. In addition, the disruption of their makeup and physical discomfort has been reported as other reasons for the helmet-wearing refusal [36]. In line with these descriptions, in another study from Iran, almost all female passengers were unhelmeted, mainly because of social norm limitations [47].

Age was also a well-known correlate of safety behaviors during driving. It is shown that middle-aged individuals are the strictest law-abiders, but the tides turn for young and especially older subjects. In fact, the elderly can even show less law-accrediting behaviors than the young [63] and are more prone to road traffic accidents [64]. The results of our study are in concordance with these findings. The reason behind the older person’s refusal to wear helmets is not discussed thoroughly in the literature, but it appears that their reduced physical and mental capacity might be imperative, necessitating further research to delineate such risk factors and propose preventive strategies.

Educational level has also been implicated as a positive correlate of helmet-wearing commitment. It is expected that people with higher educational attainments are generally more law-abide and have more positive attitudes toward safety behaviors. Likewise, possession of a driving license (where it is mandatory by law) shows the positive attitude of the driver towards the legal requirements [19]. Marital status also confers a role to decide whether or not to wear a helmet. Some authors have depicted that married individuals generally exhibit lesser risky behaviors [18, 65] and are less injured in road traffic accidents [66]. Our findings are in line with these descriptions, as married couples have had higher or at least similar helmet usage rates compared to single riders.

Authors of different studies have illustrated inconsistent findings regarding the impact of previous crashes on helmet-wearing behavior. While some might conclude that such an experience might positively affect safety behaviors, indeed, some of the included reports in our study have observed this association to be a negative one. Such higher risk-taking behavior immediately after the experience of a crash is also evidenced by other studies; however, the long-term influence of this experience on risk-taking behavior has not been proven yet [67].

The importance of helmet ownership and its association with helmet-wearing behavior is a matter of debate. In our study, this variable was of great importance in reports from low-income regions (e.g., Ghana [19] and India [26]); however, in Hung and colleagues' study [28] from Vietnam, for example, 94.6% of riders had a motorcycle helmet (regardless of using it or not). Therefore, the importance of helmet ownership should be interpreted based on the overall socioeconomic status of the region of interest and also the mechanical and technical characteristics of their manufacture. The role of motorcycle ownership was only discussed in two papers [26, 45], which was a significant determinant of helmet usage only in one [45]. Hence, reaching robust conclusions requires further research.

Another conflicting variable was religion. There is documented evidence of the association between religious beliefs with higher knowledge and attitude scores [68], avoidance of substance abuse [69, 70], and lower accident risk [69]. However, none of the included articles in our study implicated a significant role of religion in altering the attitude or decision to whether or not to wear helmets.

Moving to the characteristics of driven roads, some studies have demonstrated higher helmet usage rates on highways, while some have reported quite the opposite, i.e., helmet commitment has been higher among drivers of crowded roads in the central areas of cities. Again, the paramount place of law enforcement is clear, as some authors have proposed this as the main rationale for their observations [12, 33, 34]. In addition, the higher perceived risk of injuries on highways (due to the higher speed limits) and central areas of cities (due to the higher traffic volume) might also lead to such findings [17, 18]. A similar misperception of potential risks is also the putative underpinning for the observations of higher helmet usage among drivers of motorcycles with bigger engines. Drivers of smaller motorized vehicles (such as scooters and mopeds) generally believe that due to the lower power and speed of their vehicle, the chance of severe crashes and injuries is accordingly lower [45].

The extended dominance of law enforcement is also evident in the different helmet-wearing rates on different days of the week and hours of the day. In most reports, helmet usage was higher during the daytime and also on weekdays. The main reasonable explanations for these differences are the higher presence of police and law enforcement at these times [17, 22, 35], more recreational activities by youth at weekends [35], and difficulties in distinguishing helmet-wearing at night [22]. The frequency and the length of driven trips have had roles in deciding to use safety helmets; generally, those with more frequent and longer trips had a higher tendency to wear them. It is suggested that the importance of distance might be linked to frequency, as those who use motorcycles less frequently might also drive shorter routes [19]. The driven length is further taken as a surrogate for other variables, such as the more strict law enforcement on highways (which usually serves as the conduit for longer trips), more perceived risk for longer trips, and the lesser need to take out helmets while driving longer routes [28].

**Fig. 2:**
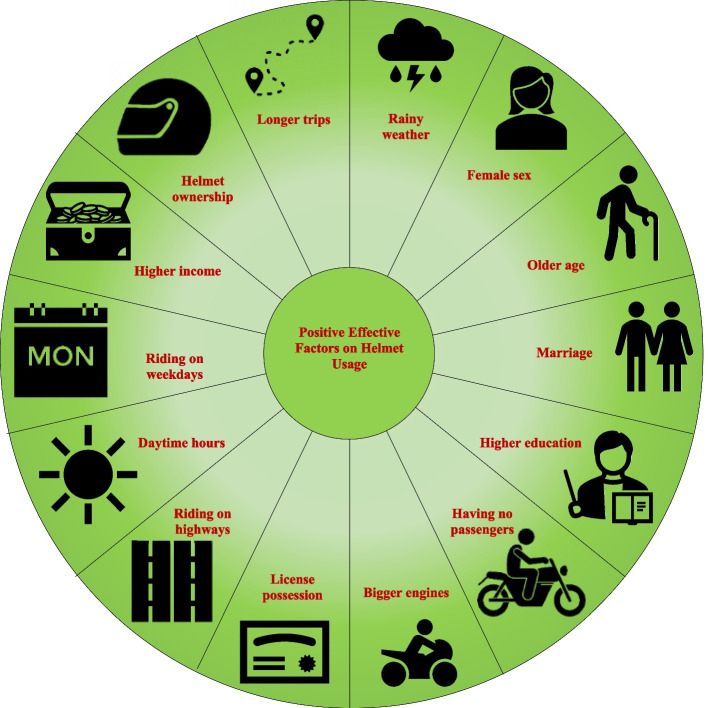
Demographic, environmental, and riding-related factors that are positive associated with motorcycle helmet usage

We also identified climate conditions, alcohol consumption, residence in urban and non-slum areas, and positive history of a previous motorcycle accident as the determinants of helmet usage in some studies; however, there was considerable heterogeneity in results, and the direction of the associations was inconsistent. Further studies are needed to decipher the exact importance of these variables. The demographic, environmental, and riding-related variables that we found as significant determinants of helmet-wearing behavior are illustrated in Fig. 2.

**Fig. 3:**
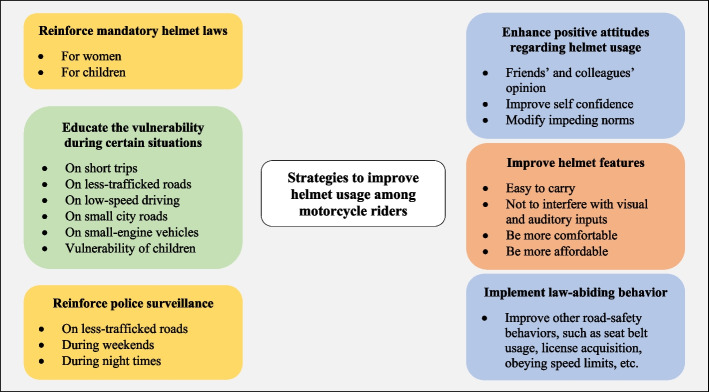
Key strategies to improve helmet-wearing commitment among motorcycle riders. These strategies are categorized into six main groups. Some factors (e.g., positive attitudes, law-abiding behaviors, and the presence and surveillance of police) are positive influencers of helmet usage. Negative factors (e.g., lower helmet-wearing behavior among women and children) will also be ameliorated by the widespread application of positive influencers

Policymakers generally put the focus of their interventions on improving the knowledge of safety behaviors to combat the issue of non-adherence to these behaviors (e.g., using safety helmets or seat belts). However, we found that even those riders who never wear helmets are aware of their benefits [18, 28, 47]. In fact, the presence of knowledge, and instead, lack of attitude, were also observed among offenders of other safety road behaviors, even in low-income and developing countries [71]. As a result, strategies should be more directed towards behavioral changes and the establishment of more positive attitudes towards safety road practices (Fig. 3).

This systematic review faces several limitations. First, the retrieved data were quite heterogeneous with respect to the target populations, type of driven motorcycles, classification of roads, educational level, income status, etc. Moreover, the included papers applied various approaches for data acquisition (i.e., observation, online questionnaire, face-to-face interviews, etc.). The publication date of studies also fell within a wide range (i.e., 1989–2021). As a result of alterations in the correct helmet-wearing protocols, motorcycle availability, safety road regulations, and motorcycle riding applications, some of the included studies might not exhibit a comparable sample compared to others. Such heterogeneities, despite the benefit of covering different situational scenarios, hindered the conduction of meta-analyses on the amassed data. Second, although none of the included studies were evaluated as low-quality, most of them lacked clear statements on sample size calculations and measures placed to mitigate bias. In fact, only seven studies had a clearly stated approach for the sample size calculation. Similarly, seven studies had declared their approaches to evaluate and manage bias, and only one study was scored for both of these criteria (Supplementary Table 3). Funding source declaration was another commonly missed factor, as half of the included studies lacked this specification. Third, for some variables (e.g., sex and type of driven roads), the reports of studies were in complete discordance, and the underpinning etiologies for such findings were not discussed thoroughly in the literature, which demands further research. Fourth, depending on the cultural, educational, and economic characteristics of different communities, the proposed strategies to enhance helmet-wearing adherence might not function effectively, and their robust benefit should be deciphered by incoming studies.

## Conclusion

In conclusion, in this systematic review, we aimed to identify factors that positively affect helmet-wearing by motorcyclists. It seems that the establishment of a stricter, more permanent law enforcement and monitoring system, along with positive alterations in the users’ attitudes toward helmet-wearing will serve as the main factors for increasing helmet usage. It is generally reported that even non-helmeted drivers agree that helmets are protective during road crashes; hence, public education should shift from this topic to more under-estimated ones. Some examples include the emphasis on the vulnerability of all motorcyclists, including adult, child, and female passengers, and the risk of serious injuries even in the low-speed ridings. Due to the interrelationship between the different types of risky behaviors, enhancing other safety behaviors (riding within the speed limit, wearing seat belts, not crossing red lights, etc.) will ultimately reinforce them all, including wearing safety helmets.

## Supplementary Information

**Additional file 1:** **Supplementary Table 1. **Search strategy for each database (searchdate: 2021/12/31).

**Additional file 2:** **Supplementary Table 2.  **Summary of the main findings of the included articles.

**Additional file 3.**

## Data Availability

The data and material used to draft this article are available via contact with the first author (PMS) or the corresponding author (VR).

## Abbreviations

RTI: Road traffic injuries
WHO: World Health Organization
CDC: U.S. Centers for Disease Control and Prevention
CENTRAL: Cochrane Central Register of Controlled Trials
PRISMA: Preferred Reporting Items for Systematic Reviews and Meta-Analyses
MeSH: Medical Subject Headings
STROBE: Strengthening the reporting of observational studies in epidemiology
OR: Odds Ratio
CI: Confidence Interval
USA: United States of America
KM: Kilometers
CART: Classification and regression tree
CCTV: Closed-circuit television
CBD: Central business district
TPB: Theory of planned behavior
HBM: Health belief model
KAP: Knowledge, attitude, and practice
