# Supplementary material for: Effective factors of improved helmet use in motorcyclists: a systematic review
Source: BMC Public Health. 2023 Jan 5;23:26. doi: 10.1186/s12889-022-14893-0 (PMC9814199; doi:10.1186/s12889-022-14893-0)
Supplement: Supplementary file 1 — Additional file 1: Supplementary Table 1. Search strategy for each database (searchdate: 2021/12/31). [file 12889_2022_14893_MOESM1_ESM.docx]

**Supplementary Table 1.** Search strategy for each database (search date: 2021/12/31).

| **Database** | **Search strategy** |
| --- | --- |
| **PubMed** | (Helmet*[TIAB] OR "Head Protective Devices"[Mesh] OR ((Protect*[TIAB] OR shield*[TIAB]) AND Head[TIAB]))  AND  ("Motorcycles"[Mesh] OR Moped*[TIAB] OR motor*[TIAB]) |
| **Scopus** | TITLE-ABS-KEY((Helmet* OR ((Protect* OR shield*) AND Head))  AND  (motor* OR Moped* )) |
| **Web of Science** | (Helmet* OR ((Protect* OR shield*) AND Head))  AND  (motor* OR Moped*) |
| **Embase** | ('helmet'/exp OR Helmet*:ab,ti OR ((Protect*:ab,ti OR shield*:ab,ti) AND Head:ab,ti)) AND ('motorcycle'/exp OR motor*:ab,ti OR Moped*:ab,ti) |
| **Cochrane library** | ID Search Hits  #1 ((Helmet* OR ((Protect* OR shield*) AND Head))): ti,ab,kw (Word variations have been searched)  #2 MeSH descriptor: [Head Protective Devices] explode all trees  #3 #1 or #2  #4 ((motor* OR Moped*)): ti,ab,kw (Word variations have been searched)  #5 MeSH descriptor: [Motorcycles] explode all trees  #6 #4 or #5  #7 #3 and #6 |
