# Supplementary material for: Effective factors of improved helmet use in motorcyclists: a systematic review
Source: BMC Public Health. 2023 Jan 5;23:26. doi: 10.1186/s12889-022-14893-0 (PMC9814199; doi:10.1186/s12889-022-14893-0)
Supplement: Supplementary file 2 — Additional file 2: Supplementary Table 2. Summary of the main findings of the included articles. [file 12889_2022_14893_MOESM2_ESM.docx]

**Supplementary Table 2**. Summary of the main findings of the included articles.

| **Study** | **Main findings^*^** | **Study** | **Main findings^*^** |
| --- | --- | --- | --- |
| Sreedharan et al. | Helmet usage was associated with female sex (OR=5.3, compared to males), positive attitude towards legislation (OR=4.5, compared to negative attitude), marital status (OR for unmarried to married=2.3), and not drunk driving (OR=3.7 compared to drunk driving). No differences for religion, educational level, or age (40> vs 40≤) were found. | Siviroj et al. | Helmet usage was associated with the type of road (lower in highway and out of town roads compared to main roads), having no passengers, earlier time of day, higher SES, older age, no history of non-helmet accosts, no history of a previous accident, and higher awareness of non-helmet wearing dangers. No associations were found for sex and type of motorcycle. |
| Fathollahi et al. | Helmet usage was associated with residence in urban vs rural areas, male sex, higher education, older age, poorer wealth index, and being single vs married. No association was found for drunk driving within the last year. | Shults et al. | Regular helmet usage was associated with less-frequent driving (OR for 1 time per year vs 2-5 times per year=2.32, 95%CI=1.17-4.62). No associations were found for sex and location of residence. |
| Aidoo et al. | Helmet usage was associated with female sex (OR for males=0.19, 95%CI=0.40-0.96), marriage (OR=2.30, 95%CI=1.33-3.96), higher educational levels (OR for tertiary=35.29, 95%CI=11.55-107.55 vs no formal school), helmet ownership (OR=20, 95%CI=8.30-48.65), and license possession (OR=3.75, 95%CI=1.9-7.42). No associations were found for age, traveled distance, history of previous accidents, riding experience, occupation, frequency of riding, history of previous accosts, and legislation awareness. | Ledesma and Peltzer 2008 | Helmet usage was associated with the female sex (OR=2.79, 95%CI=1.47-5.27), riding in the city central areas vs peripheral areas (OR for peripheral=0.40, 95%CI=0.23-0.69), riding under rainy vs good climate conditions (OR=8.09, 95%CI=3.98-16.40), and riding 250 cc motorcycles vs cross/enduro (OR for cross/enduro=0.33, 95%CI=0.14-0.79) and vs scooters (OR for scoters=0.44, 95%CI=0.21-0.95). no association was found for the time of day. |
| Babio et al. | Helmet usage was associated with higher education (OR for secondary vs university=0.63, 95%CI=0.36-1.1, P<0.01), older age (OR for >55=3.59, 95%CI=2.00-6.45, OR 35-54=1.83, 95%CI=1.28-2.83, OR for 25-34=2.30, 95%CI=1.61-3.28, compared to 16-24 as reference), average income (OR for >8800€=2.35, 95%CI=1.55-3.56, OR for 7300-8800€=1.45, 95%CI=1.01-2.08, compared to <7300 as reference), community size (OR for >180.000=2.03, 95%CI=1.46-2.82) and type of roads (OR for the city and outside roads vs only city roads=1.72, 95%CI=1.31-2.25). No associations were found for sex, marital status, employment status, duration of traffic exposure, history of previous accidents, perceived health, and smoking. | Akaateba et al. 2014 | In the total population, helmet usage was associated with the male sex (OR=1.14, 95%CI=1.04-1.25), riding inside the city (OR=1.22, 95%CI=1.14-1.31), riding during morning vs evenings (OR=1.33, 95%CI=1.23-1.45), riding on weekdays (OR=1.37, 95%CI=1.27-1.48), and being driver (OR=NR).  Among drivers, helmet usage was associated with female sex (OR for male=0.58, 95%CI= 0.52-0.65), riding inside the city (OR=1.23, 95%CI= 1.14-1.34), riding during morning vs evenings (OR=1.26, 95%CI=1.15-1.39), riding on weekdays (OR=1.57, 95%CI= 1.45-1.70), and having at most one vs at least two passengers (OR=NR).  Among passengers, helmet usage was associated with riding during evenings vs afternoons (OR for afternoon=0.30, 95%CI= 0.17-0.55). no associations were found for sex, type of roads, and the day of the week. |
| Ranney et al. | Always helmet usage was associated with female sex (OR=2.92, 95%CI=1.67-5.11), higher education (OR for at least college vs high school or less=2.56, 95%CI=1.52-4.32), no injuries from previous accidents (OR=NR), lower levels of treatment for previous injuries, and constitutes of the KAP. No associations were found for age, marital status, ethnicity, license possession, and type of motorcycle. | Pileggi et al. | Regular helmet usage was associated with having at least one close friend who always uses helmets (OR=1.29, 95%CI=1.16-1.45), male sex (OR=1.76, 95%CI=1.05-2.96), cigarette smoking (OR for not smoking=0.58, 95%CI=0.37-0.92), being driver (OR=2.92, 95%CI=2.09-4.07), and alcohol drinking (OR for not drinking=0.45, 95%CI=0.25-0.82). No associations were found for a history of previous accidents, performing sports activities, or riding over the speed limits. |
| Skalkidou et al. | Helmet usage was associated with the type of roads (OR for highways=6.25, 95%CI=4.35-9.09, and OR for suburban roads=0.67, 95%CI=0.45-0.97), riding during day hours (OR for night vs day=0.68, 95%CI=0.48-0.98), weekdays (OR for weekends vs weekdays=0.65, 95%CI=0.43-0.95), positive attitudes (OR=2.13, 95%CI=1.35-3.33) type of motorcycle (OR for 1 unit increase in engine capacity=1.52, 95%CI=1.30-1.75), driver position (OR=NR), and female sex (OR=2, 95%CI=NR). No associations were found for license possession, age, and driving experience. | Devagappanavar | Helmet usage was associated with being the driver (OR=1.72, 95%CI=1.41-2.10), riding on weekdays (OR for total population=1.27, 95%CI=1.04-1.54 and OR among drivers=1.81, 95%CI=1.37-2.38), and riding on central city roads (OR=NR). No association was found for sex. |
| Aghamolaei et al. | Constitutes of HBM. Perceived behavioral control predicted the intention to use helmets (R^2^=0.47, F=19.5, P<0.001), perceived behavioral control and behavioral intention predicted helmet usage (R^2^=0.49, F=51.7, P<0.001), and perceived barriers, self-efficacy, and cues to action significantly predicted helmet usage (R^2^=0.35, F=19.5, P<0.001). | Saeed et al. | Women with postsecondary or higher education showed significantly greater awareness regarding mandatory helmet laws (and not helmet-wearing behavior). For awareness regarding mandatory helmet legislation and belief in mandating helmet usage for female passengers, no associations were found for religion, age, marital status, and occupation status. |
| Roehler et al. | Regular, self-reported helmet usage was associated with being the driver and helmet ownership (only for child passengers). No associations were found for sex. | Bachani et al. | Helmet usage was associated with being the driver and riding during day hours. No associations were found for helmet ownership. |
| Wadhwaniya et al. | Helmet usage was associated with female sex (OR=1.8, 95%CI=1.5-2.1), older age (OR for >55=1.7, 95%CI=1.1-2.6, OR 40-54=1.9, 95%CI=1.5-2.4, OR for 25-39=1.6, 95%CI=1.3-1.9, compared to ≤24 as reference), educational level (OR for at least college vs no school=3.3, 95%CI=2.0-5.6), being driver (OR=1.8, 95%CI=1.1-3.1), engine capacity more than 100 cc (OR=1.6, 95%CI=1.0-2.4), helmet ownership (OR=40.4, 95%CI=20.8-78.5), and traveling to/from work, school, or college (OR for traveling for other activities=0.5, 95%CI=0.4-0.6). No association was found for motorcycle ownership. | Ghasemzadeh et al. | Constitutes of the TPB. Helmet usage was associated with middle/upper income (OR=46.66, 95%CI=6.16-353.18), educational level (OR for academic=44, 95%CI=5.35-361.96, and OR for high school=34.22, 95%CI=9.17-127.68, compared to elementary school), previous history of police fines (OR=2.58, 95%CI=1.05-6.31), subjective norms supporting the use of the helmet (P<0.001) and perceived behavioral control (P<0.001). no associations were found for age, license possession, occupation, or history of previous accidents. |
| Hernández et al. | Helmet usage was associated with male sex (OR=2.0, 95%CI=1.5-2.7) and the location (OR for Ibagué vs Valledupar=11.1, 95%CI=5.6-21.9). No associations were found for climate conditions, type of roads (two vs three lanes), and age (older vs younger than 59 years). | Trejo et al. | Helmet usage was associated with having no vs at least one passenger (OR=2.02, 95%CI=NR), being the driver (OR=6.52, 95%CI=NR), commercial vs private use of the motorcycle (OR=1.76, 95%CI=1.59-1.96), and residence in municipalities with higher SES. No associations were found for the time of day and the day of the week. |
| Adnan and Gazder | In univariate analysis, helmet usage before campaign implementation was associated with license possession (OR=3.06, 95%CI=1.77-5.28), a previous history of helmet stealing (OR=2.43, 95%CI=1.28-4.64), being married (OR=2.37, 95%CI=1.33-4.20), no history of a severe accident (OR=3.83, 95%CI=1.48-9.90), higher mean age (P<0.001), higher mean monthly income (P=0.0012), and older license (mean years, P=0.03). No associations were found for the mean daily number of trips, the distance of trips, mean fuel consumption, and time of day (morning and afternoon vs evening).  In univariate analysis, helmet usage after campaign implementation was associated with license possession (OR=2.73, 95%CI=1.66-4.48), older license (mean years, P=0.02), the lower mean daily number of trips (P=0.002), previous history of helmet stealing (OR=2.25, 95%CI=1.09-4.66), and positive history of a severe accident (i.e., admission at the hospital for at least three days, OR=1.75, 95%CI=1.08-2.86). No associations were found for marital status, mean age, mean monthly income, the distance of trips, mean fuel consumption, and time of day (morning and afternoon vs evening).  The combination of pre- and post-campaign data in a CART model showed that license possession is the most important determining element of helmet usage, followed by the frequency of daily trips (higher or lower, depending on license possession status), and higher age. | Ledesma et al. 2015 | In the total population, helmet usage was associated with riding on cloudy (OR=1.24, 95%CI=1.07-1.44), and rainy (OR=2.41, 95%CI=1.46-3.99) vs sunny climate conditions, riding on weekdays (OR=1.34, 95%CI=1.16-1.55), license plate possession (OR=2.33, 95%CI=1.85-2.92). No associations were found for sex, time of day, and season.  Among drivers, helmet usage was associated with female sex, having no passenger vs presence of unhelmeted (but not helmeted) passenger, motorcycle license plate possession, riding on weekdays, rainy and cloudy vs sunny climate, and the interaction between time of day and day of the week. No associations were found for the type of motorcycle, time of day, and season.  Among passengers, helmet usage was associated with female sex, helmet wearing by the driver, riding custom, touring, or sports vehicle, and motorcycle license plate possession. No associations were found for the time of day, day of the week, season, climate condition, and the interaction between the time of day and day of the week. |
| Zamani-Alavijeh et al. | Helmet usage was numerically higher among drivers and riders during winter (compared to summer), but no statistics are reported. | Conrad et al. | Helmet usage was associated with being the driver and riding on bigger roads. No association was found for sex and time of day (except for one location) |
| Khan et al. | Helmet usage was associated with belief that helmets are protective (OR=4.1, 95%CI=1.7-9.5) and passengers should wear them (OR= 3.0, 95%CI=1.8-5.0), and having at least a graduation degree (OR=2.3 95%CI=1.4-3.7), No associations were found for marital status, mean age, having children, monthly income (more or less than US$170), positive history of a previous accost, knowledge regarding helmet legislation, seeing the helmet-promoting advertisement, and knowledge regarding helmet laws. | Jiwattanakulpaisarn et al. | Self-reported helmet usage was associated with older age, male sex, more frequent riding, the more frequent presence of motorcycle checkpoints, perceived risk of accost, and positive attitude regarding helmet usage legislation for passengers. |
| Kumphong et al. | Helmet usage among all riders was associated with being the driver (OR=2.66, 95%CI=2.46-2.88), having no vs at least passenger (OR=1.55, 95%CI=1.46-1.66), engine size>125 cc (OR=1.91, 95%CI=1.71-2.15), being adult vs child (OR=2.80, 95%CI=2.23-3.53), riding during the morning (OR=5.04, 95%CI=4.74-5.370 and afternoon (OR=3.80, 95%CI=3.57-4.06) vs evenings, police presence (OR=2.22, 95%CI=2.10-2.35), absence of red light running behavior (OR for presence=0.59, 95%CI=0.52-0.65); weekdays (OR=1.11, 95%CI=1.04-1.18) and being female (OR=1.46, 95%CI=1.36-1.57) were seen among drivers; being female (OR=1.44, 95%CI=1.28-1.62) was seen among passengers. | Hung et al. | Helmet usage was associated with compulsory roads (OR=2.05, 95%CI=1.11-3.79), age (OR for >45=3.91, 95%CI=1.87-8.17, and OR for 25-44=2.22, 95%CI=1.23-4.58 vs <25 as the reference), being driver (OR=3.71, 95%CI=1.69-8.14), university or higher education (OR vs secondary or lower=3.17, 95%CI=1.62-6.23), the distance of trips>10 km (OR vs <2 km=23.29, 95%CI=8.05-67.40), positive attitudes toward universal helmet usage legislation (OR=1.93, 95%CI=1.04-3.57) and physical features of helmet use (OR=2.44, 95%CI=1.26-4.72), and engine size<50 cm^3^ vs 70-100 cm^3^ (OR for 70-100 cm^3^=0.48, 95%CI=0.13-0.74). No associations were found for the type of residence, sex, occupation, household income, riding frequency, riding experience, license possession, and alcohol consumption. |
| Ackaah et al. | Helmet usage among the total population was associated with being the driver (OR=26.92, 95%CI=17.19-42.17) riding inside the CBD (OR=1.27, 95%CI=1.10-1.46), being older than 50 years (OR=5.29, 95%CI=3.99-7.03) and 26-50 years (OR=2.66, 95%CI=2.15-3.31) vs <26 years, being male (OR=1.92, 95%CI=1.57-2.37), and having no passengers vs at least one passenger (OR=1.57, 95%CI=1.33-1.86). Male sex lost its significance among riders. all of the mentioned variables lost their significance among passengers. Helmet-wearing by the rider did not significantly affect the passenger’s helmet-wearing. | Satiennam et al. | Before CCTV enforcement, helmet usage was associated with being the driver (OR=3.41), having no vs at least one passenger (OR=1.71), riding during weekdays (OR=1.31), having engine size<125 cc (OR=2.14), and riding between 7:30-8:30 vs 6:30-7:30 (OR for 6:30-7:30=0.78). No association was found for apparent age.  After CCTV enforcement. Helmet usage was associated with being the driver (OR=3.15), having no vs at least one passenger (OR=1.64), riding during weekdays (OR=1.45), being adult vs child (OR=2.71), and riding between 7:30-8:30 vs 6:30-7:30 (OR for 6:30-7:30=0.80). No association was found for engine size. |
| Akaateba et al. 2015 | Helmet usage was associated with female sex (OR=2.92, 95%CI=1.41-6.08), marriage (OR for never been married vs married=0.44, 95%CI=0.20-0.95), formal occupation (OR for informal vs formal occupation= 0.33, 95%CI=0.15-0.71), helmet ownership (OR= 2.56, 95%CI=1.24-5.27), older age, and positive attitude regarding helmet wearing for both drivers and passengers (OR=4.83, 95%CI=2.6-9.10). No associations were found for educational level, license possession, and previous history of accost. | Xuequn et al. | Among drivers, helmet usage was associated with riding on city streets vs country, provincial, and national roads, having no passengers, and motorcycle registration. No associations were found for sex, climate conditions, day of the week, and time of day. Among passengers, helmet usage was associated with traveling on city streets vs country, provincial, and national roads, having no additional passengers, female sex, motorcycle registration, and traveling on sunny vs sunny days. No associations were found for the day of the week, the time of day, or traveling on rainy vs sunny days. |
| Mirkazemi et al. | Regular helmet usage was associated with residence in non-slum regions, belonging to a nuclear or single-parent family, male sex, and being a widow. No associations were found for educational level, age, and SES. | Sankaran et al. | Helmet usage was associated with female sex, older age (>18 vs <18 years), having a passenger (vs having no passengers), riding during morning hours (10-11:30 vs 17:30-19), and riding on weekdays. |
| Grimm et al. | Helmet usage was associated with higher education, being risk-averse, being male, traveling longer distances, having no history of previous accidents, and having higher awareness of road risks. | Merali et al. 2020 | Helmet usage was associated with being taxi drivers vs non-taxi riders (OR=1.49, 95%CI=NR) and riding on non-residential vs residential roads (OR=1.39, 95%CI=NR). |
| Kulanthayan et al. | Helmet usage was associated with riding inside towns (OR=3.51, 95%CI=2.25-5.48), being>21 years (OR=1.75, 95%CI= 1.05-2.90), being female (OR=1.70, 95%CI=1.06-2.71), traveling a distance>2 km (OR=3.74, 95%CI=2.35-5.94), and predicting the presence of police (OR=2.20, 95%CI=1.21-4.00). No associations were found for educational level, type of license, riding experience, and type of helmet. | Gkritza | Among drivers, helmet usage was associated with the time of day (7:00-10:00 vs 10:00-15:00 and 15:00-18:00), riding on primary and secondary vs city roads, riding on rainy vs cloudy and rainy/cloudy vs sunny days, the presence of helmeted vs unhelmeted passenger, and riding during April vs August.  Among passengers, helmet usage was associated with the time of day (7:00-10:00 vs 10:00-15:00 and 15:00-18:00), the presence of helmeted vs unhelmeted drivers, and riding on rainy vs cloudy and rainy/cloudy vs sunny days. No associations were found for the type of road and month of the year. |
| Papadakaki et al. | Helmet usage was associated with female sex, higher education, lower consumption of high-concentrated alcoholic beverages, riding between 14 to 22, riding towards home, work, or school, and positive history of a previous accident. No associations were found for age, mean driven distance, and season. | Grummon et al. | Helmet usage in all youth was associated with parental supervision (in total but not age-specific subgroups), perceived severity, and self-efficacy in wearing helmets (in total but not age-specific subgroups). No associations were found for parental reminding (not for the 9-12 years group, which was significant) and monitoring, perceived susceptibility, and response efficacy in wearing helmets. |
| Dandona et al. | Helmet usage was associated with younger age, female sex, higher education, and riding motorcycles, scooterettes, and scooters, compared to mopeds, and motorcycle ownership. | Li et al. | Helmet usage was associated with riding on principal roads vs lateral streets, riding during mornings vs evenings, and riding on weekdays (all Ps<0.001). |
| Fletcher et al. | Helmet usage was associated with older age (OR for one year increase=1.05, 95%CI=1.02-1.09), engine size>150 cm^3^ (OR=2.72, 95%CI=1.11-6.65), and driving status (only in univariate analysis). No associations were found for license possession, occupation (employed vs not employed), and insurance possession. | Jomnonkwao et al. | In rural areas, helmet usage intention and behavior were associated with perceived severity, cue to action, and perceived benefits. . No associations were found for other constructs of HBM, namely health motivation, perceived barriers, and perceived susceptibility.  In urban areas, helmet usage intention and behavior were associated with health motivation. No associations were found for other constructs of HBM, namely perceived susceptibility, perceived severity, perceived benefits, perceived barriers, and cue to action. |
| Siebert et al. | Helmet usage was associated with being the driver (OR=NR). | Olewinski et al. | Helmet usage among student-athletes was associated with the coach’s rule. |
| Adewoye et al. | Helmet usage was associated with older age (OR for 40-49 years=2.93, 95%CI=1.37-6.25 and OR for 30-39 years=2.14, 95%CI=1.18-3.88; vs 20-29 years), being married (OR=2.07, 95%CI=1.19-3.60), and having a tertiary degree (OR vs secondary degree=2.55, 95%CI=1.28-4.21). No associations were found for religion and belonging to a tribe. | Merali and Bachani 2018 | Helmet usage among child (<12) passengers were associated with driver helmet usage (OR=6.18, 95%CI=5.11-7.47), riding on Sunday vs Saturday (OR=0.63, 95%CI=0.48-0.81), Friday (OR=0.80, 95%CI=0.64-0.98), and Tuesday (OR=0.82, 95%CI=0.67-0.99), riding at noon (OR=1.255, 95%CI=1.06-1.48) and 17:00 (OR=1.24, 95%CI=1.05-1.44) vs 9:00 (but not 19:00 vs 9:00), lower total passengers (OR for higher mean=0.45, 95%CI=0.40-0.51), and riding in the capital province vs other 4 provinces. No association was found for 1 vs 2 or more child passengers. |
| Babazadeh et al. | Helmet usage was associated with Medium and higher vs minimum or low income (OR=46.66, 95%CI=6.16-353.18) and having university (OR=44, 95%CI=5.35-61.96) and high school (OR=34.22, 95%CI=9.17-127.68) vs primary degree, and previous history of police fines (OR=2.58, 95%CI=1.06-6.31). no associations were found for age, occupation, license possession, and previous history of accidents. | Bao et al. | The common reason for helmet usage was to provide protection against injuries and to avoid police fines. No statistics are provided regarding the association of helmet usage with the time of day, day of the week, or age. |

*In studies with both univariable and multivariable analyses, only the results of the latter one are considered.

OR, odds ratio; CI, confidence interval; vs, versus; KAP, knowledge, attitude, and practice; NR, not reported; CART, classification and regression tree; SES, socioeconomic status; km, kilometers; TPB, theory of planned behavior; CCTV; HBM, health belief model.
